# Magnetic Stirring May Cause Irreproducible Results in Chemical Reactions

**DOI:** 10.1021/jacsau.5c00412

**Published:** 2025-06-11

**Authors:** Vera A. Cherepanova, Evgeniy G. Gordeev, Valentine P. Ananikov

**Affiliations:** Zelinsky Institute of Organic Chemistry, 68429 Russian Academy of Sciences, Leninsky Prospect, 47, Moscow 119991, Russia

**Keywords:** reproducibility, organic synthesis, catalysis, nanoparticles, magnetic stirrer

## Abstract

Magnetic stirrers, the most widely used and ubiquitous devices for performing chemical reactions in laboratory settings, may cause reproducibility problems. Reproducibility in a range of chemical processes can be affected by various factors, ranging from minor to significant effects, including yield, composition, and glassware contamination. In this study, we illustrate the reproducibility issues that may arise from the use of a magnetic stirrer for three fundamental types of chemical reactions. Significant differences were found in the reaction rates and sizes of the nanoparticles obtained via parallel synthesis with the same magnetic stirrer. For catalyst preparation, differences were observed in the morphology of the metal nanoparticles and the process rate depending on the location of the reaction vessel on the magnetic stirrer. In the case of organic synthesis examples, the conversions of parallel catalytic cross-coupling reactions in vessels standing beside each other on the same magnetic stirrer can be significantly different. The results of these experiments revealed the influence of previously unaccounted-for factors, and here, we suggest a control experiment to improve reproducibility. Given the ubiquitous use of magnetic stirrers in chemistry, biology, life sciences, and material sciences, the revealed reproducibility-affecting factor is of broad concern.

## Graphical abstract

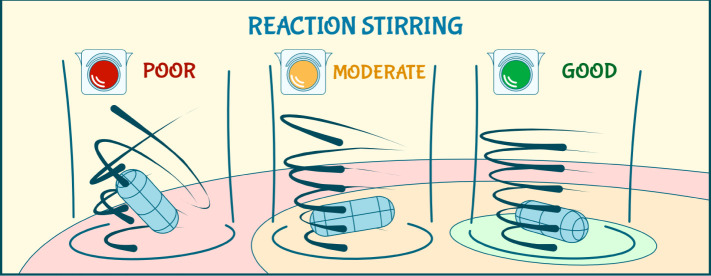

## Introduction

Chemical synthesis is a key technology in modern research facilitated by progress in the development of new methodologies^1–3^ and the optimization of reaction processes.^4–8^ The issues of new methodology development are becoming particularly relevant in light of the growing discussions on the problems of reproducibility of reported results in scientific research.^9–11^ Recently, a plethora of articles have highlighted the so-called reproducibility crisis, urging researchers to meticulously reevaluate numerous studies.^10–16^ The underlying causes of these reproducibility challenges are multifaceted (Figure 1). They range from questions surrounding the integrity of research and the rapid pace of publishing to statistical inaccuracies.^17–22^ Of paramount concern in practical chemistry are the errors or inconsistencies that arise during the design and execution of experiments. Such errors can render experiments poorly reproducible (or even nonreproducible) when attempted in different laboratories, underscoring the importance of careful technique setup and a comprehensive understanding of the experiment at hand (Figure 1). While factors such as variability in solvent quantity, temperature fluctuations, catalyst loading, and reactant ratios have received scholarly attention,^23–27^ others remain elusive and are often overlooked by researchers. Specifically, the configuration of vessels,^28,29^ heat and mass transfer within the reaction mixture,^29–33^ challenges associated with thoroughly mixing the reaction media,^34–36^ the purity of reactants and intermediates,^17,37,38^ and variations in methods^39^ can profoundly influence reactions. A notable source of error is the contamination of reagents^9,40,41^ and phantom reactivity^42–44^ caused by impurities. Even trace amounts at the ppm/ppb level have been reported to significantly affect sensitive reactions.^43–45^ A brief illustrative overview of the primary factors contributing to reproducibility issues, their associated risks, and their estimated probabilities of occurrence is depicted in Figure 1 and detailed in Table S1.

**Figure 1:**
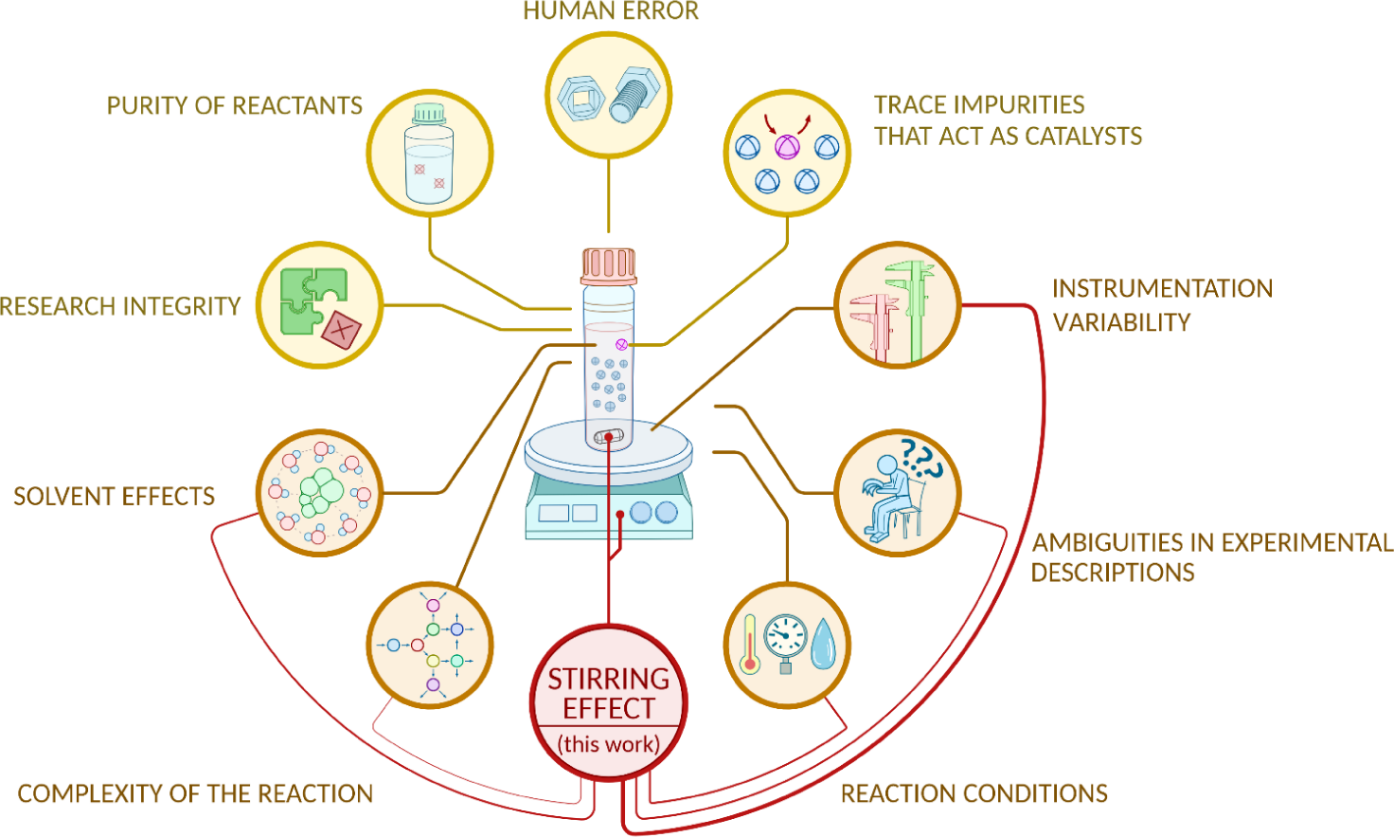
Critical factors affecting chemical reactions. Schematic overview of reproducibility issues in stirred reactions. The magnetic stirring effect emerges as a critical multifactorial variable, interacting with (i) reaction conditions, (ii) solvent properties, (iii) instrumentation variability, (iv) experimental descriptions, and (v) the complexity of the reaction.

In our extensive research, we consistently observed discrepancies in the reproducibility of both the yields and selectivity of catalytic organic synthesis reactions, as well as in the size and distribution of nanoparticles synthesized during catalyst preparation. A deep exploration of the root causes of these reproducibility issues in catalytic reactions and catalyst formulation led us to an unexpected finding related to stirring practices on standard magnetic stirrers, which are the backbone of contemporary laboratories. Astonishingly, we found that the outcomes of the reactions were significantly influenced by unforeseen variables linked to magnetic stirring. Importantly, the nonreproducibility factor highlighted in our work interplays with several other elements tied to experimental design, reaction processes, and procedural documentation (Figure 1).

Our findings highlight a critical oversight, one that researchers might not yet recognize, which may significantly impact chemical reactions, catalysis, and nanoscience. Not limited to the reported reactions and considering the ubiquitous presence of magnetic stirrers across chemistry, biology, and pharmaceutical laboratories (to name just a few fields), the nonreproducibility factors elucidated in this study warrant keen attention. Such factors could potentially influence a broader spectrum of reactions beyond the synthesis, catalysis, and nanoscience examples shown here. The potential vulnerabilities introduced by this overlooked factor could be profound, especially if researchers remain uninformed. We assert that these findings merit broad dissemination within the research community.

## Results and Discussion

The magnetic stirrer is a traditional and universal tool widely used in research laboratories and has the ability to perform parallel execution of numerous reactions. Typically, uniform stirring and mixing are taken for granted via this widespread experimental technique. In this research, we note that the nature of the bar rotation changes when it is placed at different positions on a magnetic stirrer, ceasing entirely at specific locations on the stirrer plate.

To illustrate the variability in bar rotation across the stirrer surface, standard vials were selected. Owing to their compact size, these vials can be accommodated in substantial numbers on a magnetic stirrer, and this situation routinely occurs during experimental optimization. For geometrical quantification, a stand was developed to secure the vials both horizontally and vertically in relation to the center of the stirrer surface. This design enabled multiple reactions to be carried out in parallel at fixed positions. The stand was set along the diameter of the stirrer plate. Geometrical locations at the first level correspond to room-temperature stirring or stirrer-heating processes. The positions at the upper level correspond to the vessel locations when heating with a water/oil bath is utilized (the reaction vessels are placed above the stirrer surface). Considering regular research in chemistry, various locations of reaction vessels are quite common in laboratories (see Supporting Information section “Some real-life examples of magnetic stirrer usage”).

### Preparation of Pd/C Catalyst Material

To demonstrate the discrepancies introduced by the spatial reaction vessel location, the decomposition of the zerovalent palladium complex Pd_2_dba_3_·CHCl_3_ in the presence of a carbon material (nanotubes) was chosen as a model reaction for producing highly popular Pd/C catalysts. This catalyst formation is easy to monitor because of the distinct correlation between the solution color change and the rate of Pd deposition from the solution to the surface. The resulting Pd/C material is a heterogeneous catalyst, which holds potential for application in a myriad of catalytic reactions.^46–48^ Multiwalled carbon nanotubes (MWCNTs) were used as an example of high-performance carbon support for heterogeneous catalysts.^49^

As a dedicated experimental setup, to contrast the processes in systems situated at varying positions on the stirrer, a vial stand was manufactured by using 3D printing (Figure 2b). This stand is equipped to simultaneously position 15 vials across three levels, arranging them from the center to the periphery of the stirrer’s surface. Evolutions in characteristics over time were systematically recorded using time-lapse photography, NMR monitoring, and transmission electron microscopy (TEM) of the precipitates.

**Figure 2:**
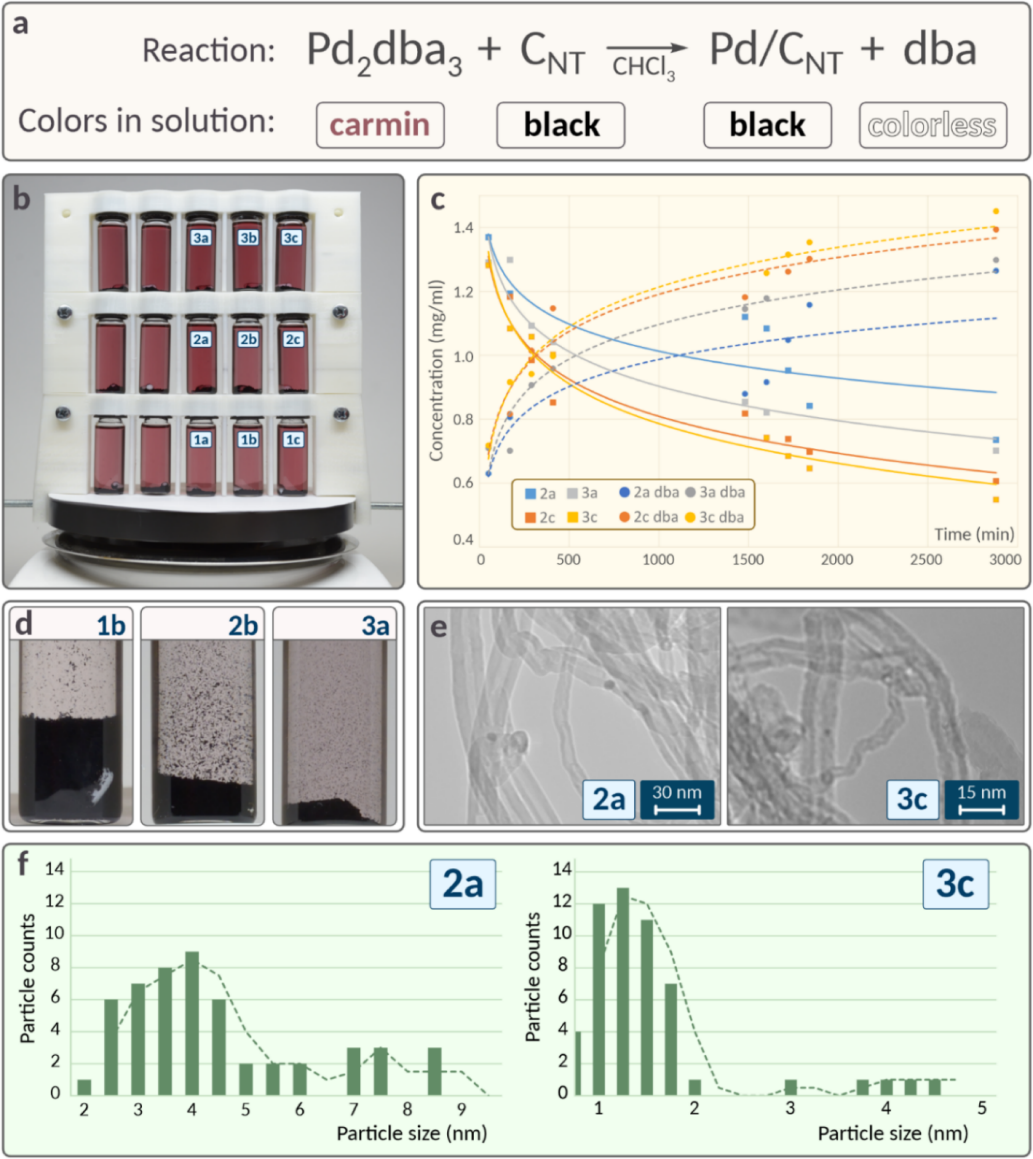
Experimentally measured results in the preparation of Pd/C catalyst: (a) reaction equation for Pd/C catalyst synthesis with colors of each component; (b) experimental stand showcasing vials on the magnetic stirrer (vials are marked as 1a–1c, 2a–2c and 3a–3c; corresponding marks are indicated on other pictures); (c) ^1^H NMR monitoring of the reaction: blue solid and dotted lines represent Pd_2_dba_3_ and free dba concentrations for the 2a position, orange solid and dotted lines represent Pd_2_dba_3_ and free dba concentrations for the 2c position, gray solid and dotted lines represent Pd_2_dba_3_ and free dba concentrations for the 3a position, yellow solid and dotted lines represent Pd_2_dba_3_ and free dba concentrations for the 3c position; (d) the height of the carbon material pillar and the solution color in differing vials after stirring; (e) TEM images of resulting palladium nanoparticles deposited on nanotubes for the 2a and 3c samples; (f) particle size distribution for the representative systems.

The time-lapse images captured disparate rates of discoloration among the systems. Given that the solution color can serve as visual evidence for the complex decomposition rate (Figure 2a) and, consequently, catalyst synthesis (see Supporting Information, the subsection “Factors changing in systems with different locations”), the rate of discoloration emerges as a visualization of the Pd/C formation process for different positions of the vials (Figure 2b).

NMR monitoring provided an independent, quantitative characterization of the differences revealed visually. The reaction kinetics of the four systems are shown in Figure 2c. It should be noted that several factors may additionally cause strong differences in the decomposition rates of the studied reaction; for example, side processes (i.e., dba precipitation on the carbon material) may begin to occur, which may affect the overall picture.

Although the initial complex concentrations are the same, they are noticeably different by the end of monitoring at different positions, indicating different kinetic parameters for an identical reaction but at different positions of the system on the stirrer.

The variability in the reaction kinetics, which is based on the position of the reaction vessel on the magnetic stirrer, poses distinct ramifications to consider. These include mistakes in defining system kinetic parameters, uneven product morphology, difficulties in comparing reactions on the same stirrer, and problems with replicating reactions when changing the vessel position, among others.

To explain the observed reaction rates, several factors must be considered: the intrinsic rate of decomposition for Pd_2_dba_3_ (see the “Formation of metal nanoparticles” section) and the influence of the carbon material, particularly its changes during stirring (Figure 2d).

In different vials, visual changes in the volume of carbon nanotubes where deposition occurred were also noted (Figure 3a,b). This phenomenon is likely attributable to the grinding of the carbon material. When a vessel is positioned away from the central axis of the stirrer, the magnetic bar tends to gravitate toward this axis. As a result, the bar aligns in an inclined manner, leading to persistent contact with not only the base of the vessel but also its walls. Such bar movement may grind the carbon material, causing it to settle more loosely post-stirring (Figures 3b and S20). Grinding of carbon nanotubes may also modify their catalytic activity by potentially increasing their specific surface area and possibly inducing metal leaching.

**Figure 3:**
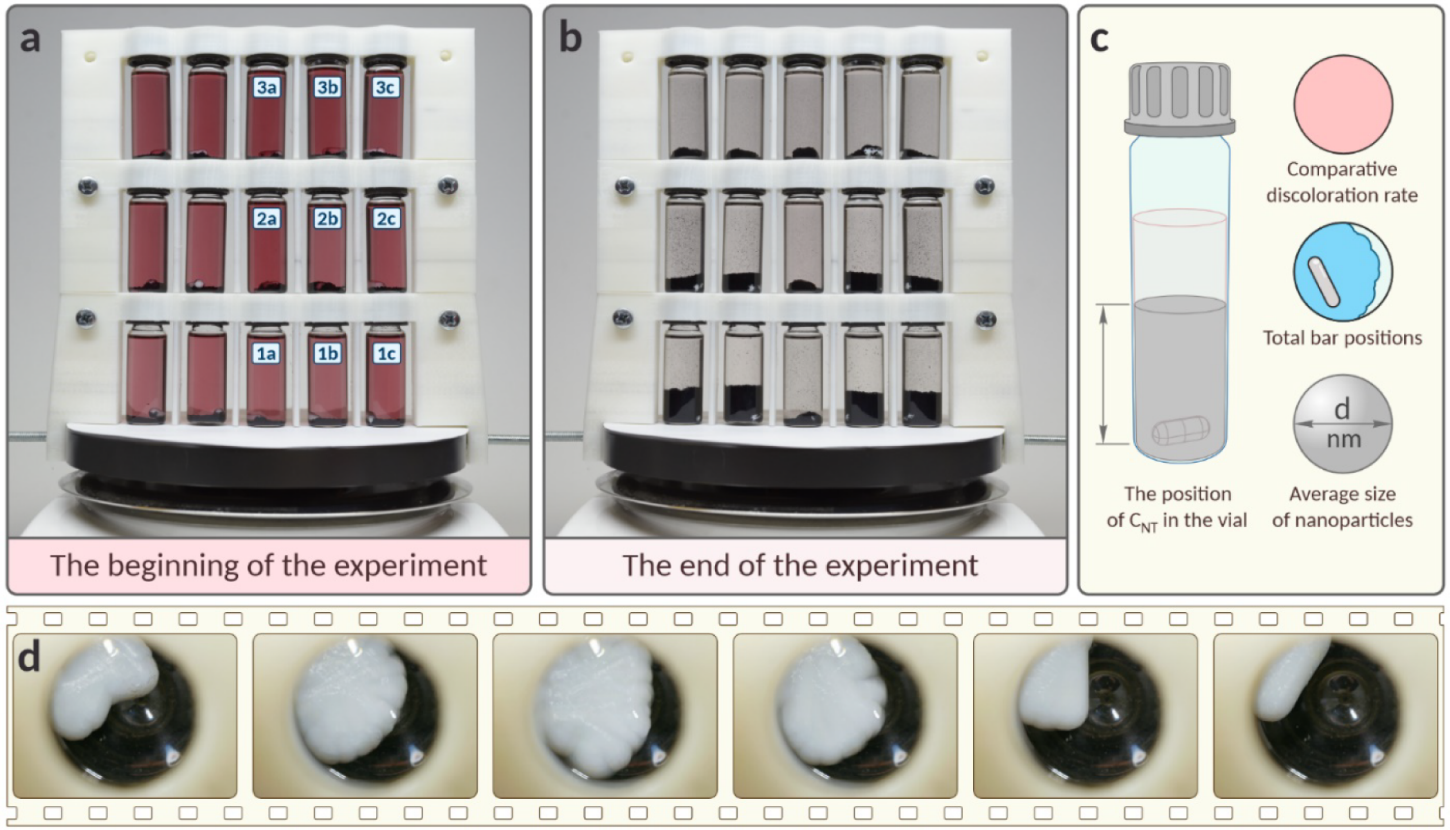
Variations in the parallel reactions. Photos of the Pd/C synthesis setup at the beginning (a) and end (b) of the experiment and the parameters used to describe the systems (c). (d) Cumulative representation of the bar positions at location 3c. Each image is composed of 100 frames captured within 1.67 s. The first frame was taken 55 s after stirring initiation.

The observed variations in the state of the nanotubes prompted an investigation of the catalyst using electron microscopy. Images captured via transmission electron microscopy enabled the determination of the sizes of the deposited metal particles (Figure 2e). Statistical analysis of each sample revealed that the average particle size could deviate by more than double within systems located on the same stirrer (Figures 2f and S19).

We performed a detailed analysis and revealed the following factors: the vial position relative to the magnetic stirrer center, which was determined both horizontally (marked by a letter in this study) and vertically (marked by a number in this study); the rate of discoloration, which is an indicator of the reaction proceeding; the nanotube column height at the end of the reaction and their grinding; the average size of the nanoparticles; and the bar position in the vial (Figure 3c). The latter turned out to be key, as illustrated by the example of a bar in a vial at position 3c (Figure 3d). The image is composed of 100 frames taken over a period of 1.67 s. The first frame was taken 55 s after the start of stirring. Even with this short observation time, we observe the bar stopping for more than 1.5 s. The position of the bar in the vessel also clearly depends on the type of bar used. As we moved away from the center, we noted distinct differences in the movement patterns of the different stirrers. Furthermore, the size of the bars affects the reaction kinetics. These effects are described in more detail in the Supporting Information (see the section “Comparison of different stir bar sizes and shapes”).

As a result of time-lapse observations of the system of 15 vials, the solutions at positions 1a and 1b were the first to discolor, with position 1c following shortly after. While the solutions in positions 3a and 2a were the last to discolor. In between, the other ones were discolored. This pattern suggests a significant decrease in the reaction rate for all of the positions above the stirrer surface.

Upon completion of the Pd/C synthesis, TEM analysis revealed differences in the size of the palladium particles deposited on the nanotubes in the different systems (Figure S19 a−i), which may affect the catalytic activity of the resulting catalysts. The largest particle size was observed in the system with the longest discoloration time; in the first two systems, the particle sizes coincided within the accuracy of the discoloration rate. Furthermore, differences in the sizes of metal nanoparticles synthesized simultaneously on a single magnetic stirrer were confirmed for Cu/C synthesis (Figure S21 and Table S2).

At the end of the reaction, visual differences were observed in all vials – at 1b, 1c, 2b, and 2c pillars of nanotubes, which were larger in volume than the initial volume of the loaded carbon material. At the lower level, the height of the columns is greater than that at the second level. The observed pillar heights might be related to the varying bar movements during stirring and subsequent grinding of the nanotube coils, resulting in a more dispersed arrangement.

These factors are described in more detail in the Supporting Information (see the subsection “Factors changing in systems with different locations”).

### Formation of Metal Nanoparticles

To ascertain the generalizability of these observed stirrer-induced differences, an additional system involving the formation of Pd nanoparticles in solution was examined without a carbon material (Figure 4a). In this system, palladium nanoparticles with specific morphologies were formed during the reaction.

**Figure 4:**
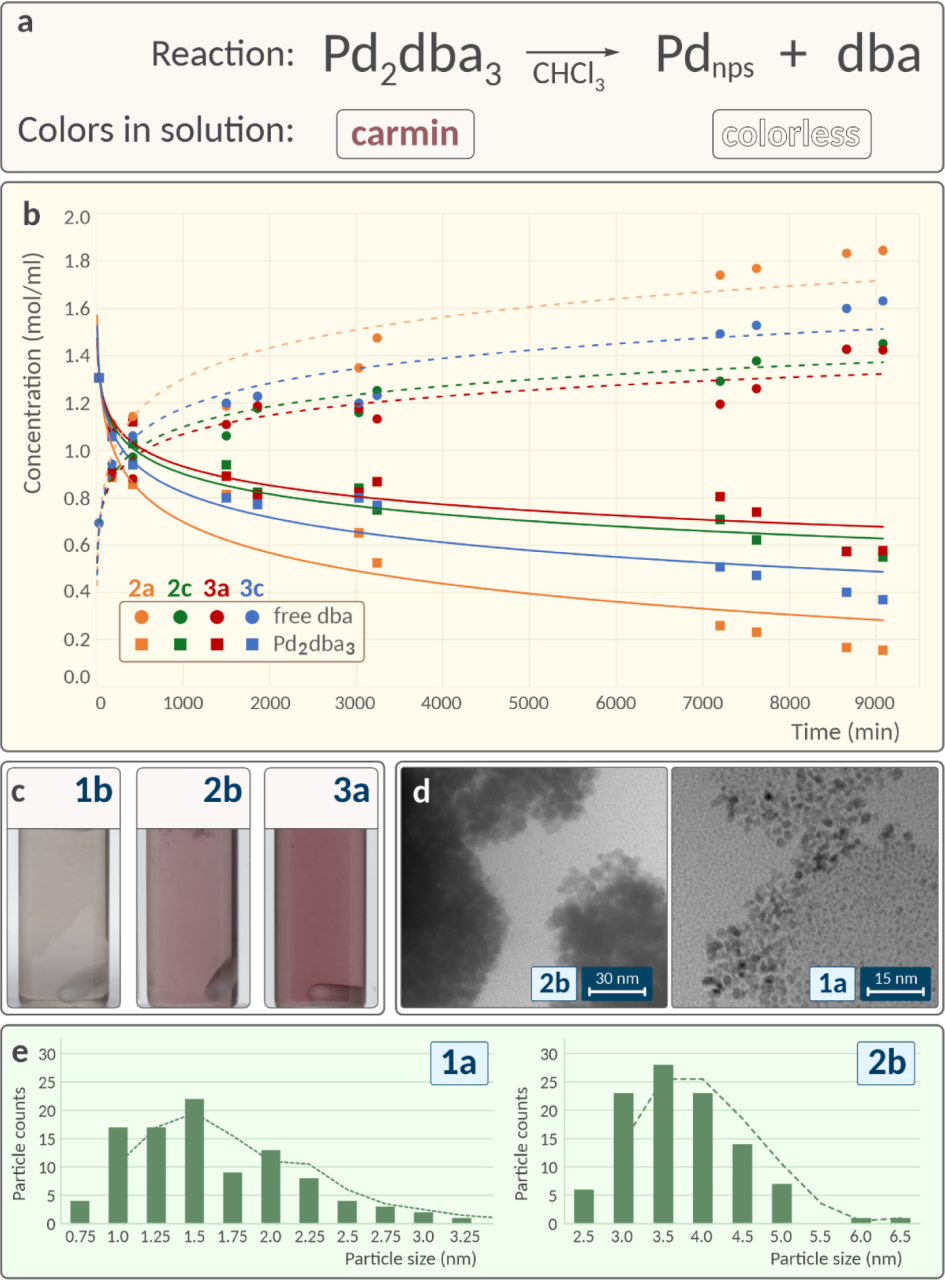
Illustration of variations during the preparation of Pd nanoparticles in solution: (a) reaction equation for Pd nanoparticles synthesis; (b) ^1^H NMR monitoring of the reaction: orange solid and dotted lines represent Pd_2_dba_3_ and free dba concentrations for 2a position, green solid and dotted lines represent Pd_2_dba_3_ and free dba concentrations for 2c position, red solid and dotted lines represent Pd_2_dba_3_ and free dba concentrations for 3a position, blue solid and dotted lines represent Pd_2_dba_3_ and free dba concentrations for 3c position; (c) variation in solution color across different vials at a specific time point; (d) TEM images of palladium nanoparticles from different samples; (e) comparison of particle size distributions between two distinct systems (see Figure 3a for denoting the locations of the vessels).

Palladium nanoparticles were synthesized using the vial stand as in the prior experiment (Figure 3a), which included five vials in a linear arrangement, with the central vial aligned on the main axis and spanning three vertical levels.

Time-lapse photography tracked visual changes in the systems until full discoloration, indicating that the reaction progressed to at least 95% completion. Without the presence of a carbon material in this setup, the reaction dynamics shifted substantially. The influential factor, a developed surface with numerous active centers assisting in metal adhesion and complex breakdown, was absent. Consequently, the duration of the experiment greatly increased due to a marked reduction in the complex decomposition rate, especially given the lack of surfaces other than the vial walls.

Distinct discoloration rates were evident among the systems, a disparity accentuated without the interference of suspended carbon particles (Figure 4c). NMR monitoring from the noted positions substantiated these rate disparities (Figure 4b). Unlike systems with carbon material, in this case, no carbon material surface-related processes are observed, owing to which the comparative reaction rate changes and the dependencies themselves become clearer, demonstrating the differences between the positions.

Upon the conclusion of the reaction, the grids were introduced into continuously stirred vials for TEM analysis (Figure 4d). Subsequent microscopy studies revealed significant differences in the particle size distributions, even among adjacent vials (Figure 4e). Mirroring the previous experiment, achieving palladium nanoparticles with a specific morphology proved challenging when multiple reactions were conducted simultaneously on a stirrer.

### Stirrer Effect on the Catalytic C–C Coupling Reaction

To explore the effects on the catalytic reactions, we chose the Suzuki–Miyaura cross-coupling reaction, which is a valuable tool in research and industry for carbon–carbon bond formation.

As in the previous experiments, the reactions were carried out in vials with new bars to avoid traces of catalytic metal. To accommodate multiple simultaneous reactions, a custom stand was designed, allowing for nine reactions. This design ensures that all of the vial bottoms are in contact with the stirrer surface (Figure 5b). The reaction was carried out at 70 °C with a commercial Pd/C catalyst (Figure 5a). In this reaction, we employed a design that is typically used for organic synthesis and catalysis, utilizing a water/oil bath for heating.

**Figure 5:**
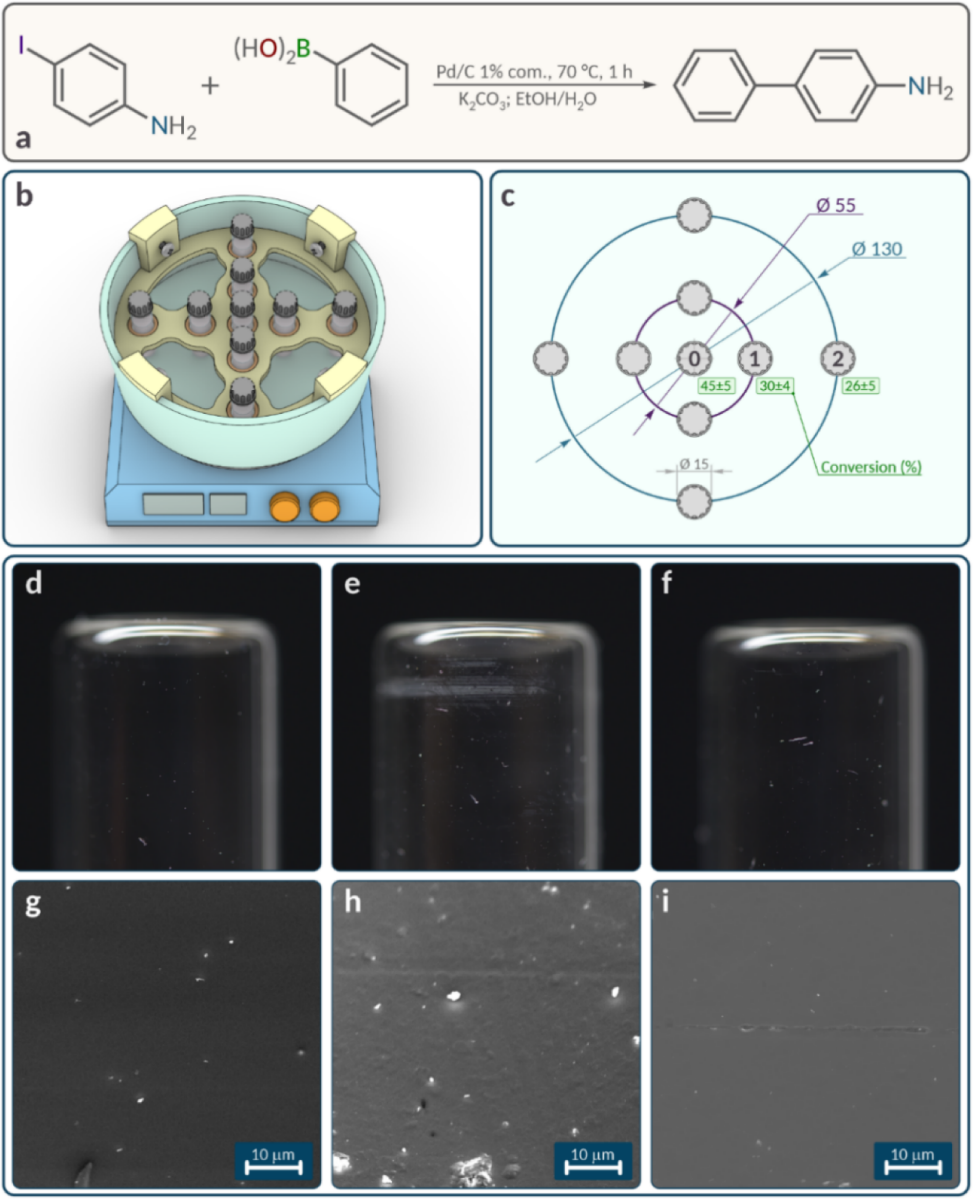
Overview of reaction conversions and vial placement: (a) reaction equation for the catalytic C–C coupling reaction; (b) positioning of vials on the stirrer surface; (c) vials positioning scheme in relation to the stirrer surface with stand dimensions and reaction conversions (%) in them; (d)–(f) vial macrophotographs: standard, after Pd nanoparticle synthesis, and after Pd/C catalyst synthesis, respectively; and (g)–(i) vial TEM images: standard, after Pd nanoparticle synthesis, and after Pd/C catalyst synthesis, respectively.

To monitor differences in the catalytic systems, ^1^H NMR spectroscopy was used to determine the conversion in all the systems simultaneously after 1 h. As observed experimentally, the central position presented the highest conversion (45%). A noteworthy drop in conversion was observed for the intermediate positions, despite their relatively short distance from the center (27.5 mm between the vial centers). For the system that was closest to the central system, the decrease in conversion was 15%. The outermost vials presented conversion rates comparable to those of the intermediate vials, an unexpected outcome given the greater distance between the outer and intermediate vials (Figure 5c). This experiment underscores the challenge of ensuring reproducibility when the placement of the reaction vessel deviates from the surface of the magnetic stirrer and its center.

To gain deeper insights into the disparities stemming from varied placements on a magnetic stirrer, we turned our attention to magnetic bar movements. If the reaction vessel is shifted from the stirrer center, the magnetic bar moving pattern changes, ceasing completely at a certain distance. We captured this rotational behavior on video, and the frames were superimposed on each other to simplify the presentation of the results (see Supporting Information section “Magnetic bar motion dynamics”).

When the bar rotates without restrictions, its cumulative position forms a circle-like pattern. In contrast, oscillating movements produce an hourglass or fan shape, whereas a stationary bar results in a single static image.

A critical observation from these images is the periodic cessation of bar movement, which is especially prominent in the system farthest from the stirrer’s center. In this specific setup, the bar remained immobile for more than 35% of the observed duration once acceleration was factored out. Comparable but briefer halts were noted in other systems. The nature of the movement depending on the height relative to the stirrer surface is described in more detail in the Supporting Information “Effect of geometrical position on the stirrer”. Such interrupted rotation and chaotic bar motion are likely to compromise effective stirring, disrupting solution homogeneity, and overall system consistency. Consequently, this erratic stirring mode not only delays reactions but also introduces unpredictable kinetics, rendering several parameters random.

As an additional point to consider, the movement patterns of bars can change when different bar types are used, as well as when the vessel type is varied. Detailed differences are shown in the Supporting Information sections “Comparison of different stir bar sizes and shapes” and “Reactions in different vessels”. These observations show that a combination of position-dependent stirring and vessel shape indeed may have a more complex effect on reaction reproducibility.

### Mechanical Effects and Damage to the Reaction Vessels

In our investigations regarding bar movement during stirring, it was observed that at certain off-center positions, the bar frequently made contact with the vial walls. Given this observation and considering that the increase in carbon nanotube volume might be attributed to bar-induced breakage of the material, we anticipated potential mechanical scratching or damage to the vials.

To validate this hypothesis, three new vials were selected for experimentation. The first one served as our control; the second was filled with a reaction mixture for Pd nanoparticle synthesis, and the third was used for Pd/C catalyst synthesis. Based on our earlier observations that off-centered systems experienced bar friction against the vial walls, we intentionally positioned these vials in off-center locations on the stirrer. The vials were then subjected to a continuous 35 h stirring process. Next, the samples were cleaned with acetone, dried, and prepared for examination. Photography was utilized to capture the macrolevel details, with the vials secured in place using a vice (Figure 5d–f). For a detailed microscopic view, small fragments of the vials were taken and analyzed by scanning electron microscopy (Figure 5g–i).

As expected, the control vial lacked any defects at both the macro- (Figure 5d) and microscale (Figure 5g). Our findings revealed the presence of cracks and other forms of surface deformities on the other two vials, spanning both macro- and microscales. A point of intrigue was the more noticeable damage to the vial devoid of nanotubes (Figure 5e,h) in comparison to that of its counterpart containing them (Figure 5f,i). This suggests that while the carbon nanotube material is destroyed due to bar friction, it might also play a role in mitigating the oscillatory impacts of the bar.

However, the emergence of even minor abrasions over such a brief duration raises concerns regarding the long-term integrity of glassware, especially given that typical operational lifespans are far longer than a few tens of hours. The accumulation of impurities in the scratches and their transfer to another reaction is a serious concern, well-known for “pseudo-Pd-free” reactions and phantom reactivity.^39,42^

### Improving Reproducibility

The solution we propose is to perform a control experiment with a single reaction vessel placed in the most appropriate position (usually at the center of a magnetic stirrer) and to report the results in publications. This approach was validated by our observations across various reaction types, including heterogeneous, homogeneous, and metal-free systems (see Supporting Information section “Reproducibility screening in different reactions”).

Some experiments may involve optimization with many parallel reactions with one stirrer. This may be performed for the optimization of the reaction conditions. However, once the conditions are optimized, the final experiments reported in this work should involve a single reaction vessel placed in the most appropriate position at the stirrer. This experimental verification is easy to perform and can be done in any lab worldwide. This control experiment solves the problem related to the irreproducibility of magnetic stirring.

The following recommendations are proposed:

- for the final reported yields under optimized conditions, a relevant number of control experiments should be performed with a single vessel placed in an ensuring complete stirring position.
- for the optimization of the reaction conditions, if the deviations between the result obtained via parallel execution and the control experiment proposed in this manuscript are significant, the researchers will be warned and can repeat important optimizations in the same way as the control experiment is done.

Among the other issues to consider, to achieve efficient stirring along with reproducible results, the following conditions should be met. For similar experiments, the same stirrer type/model, stirring parameters (rate, temperature), geometric positions of the reaction vessels, stirring bar size/type, and vessel size/type are recommended. All of the parameters should be documented in the experimental procedures. Notably, this is typically *never done* in modern experimental studies. For full reproducibility, the reaction setup should be photo/video-recorded, and stirring positions should be preserved.

Despite the varying sensitivity of reactions to changes in stirring, the optimum position of the reaction vessel is in the center of the stirrer, as this is the only way to avoid damage to glassware and minimize contamination effects (impurities accumulate quickly in glassware defects and cannot be easily washed out). This is also confirmed by magnetic field calculations on the stirrer surface, as well as its interaction with the magnetic bar. When deviating from the central position, the motion of the bar becomes unstable (see Supporting Information section “Modeling of the interaction between magnets by finite element method”).

Without calculations, the “green” zone with optimal stirring efficiency can be determined by defining the central position between the magnetic poles and the bar length via any convenient method (see Supporting Information section “Effect of geometrical position on the stirrer” and Figures S26–29). In Figure 6a, small granules of ferromagnetic materials are scattered across the stirrer surface. At the magnetic poles, the metal particles line up perpendicularly to the stirrer surface, framing an area within which the magnetic field differs only minimally. On the rest of the surface, the particles line up along lines of force, and their density is directly proportional to the magnitude of magnetic induction (Figure 6b–f). Such a visualization of magnetic fields makes it possible to determine the locations of the magnetic poles and the distances between them. We have also proposed a simple procedure to detect various stirring efficiency zones on the stirrer surface for any given magnetic stirrer, vessel, or bar (Figure 6g and Supporting Information section “Effect of geometrical position on the stirrer”). Placing reaction vessels in the “green” zone is most appropriate for optimal reproducibility, and installation in the yellow zone on the stirrer surface is acceptable for rough routine optimizations of many reactions in parallel. At the same time, the red zone on the stirrer surface, as well as a distance along the vertical axis, is most susceptible to possible irreproducibility (Figure 6g).

**Figure 6:**
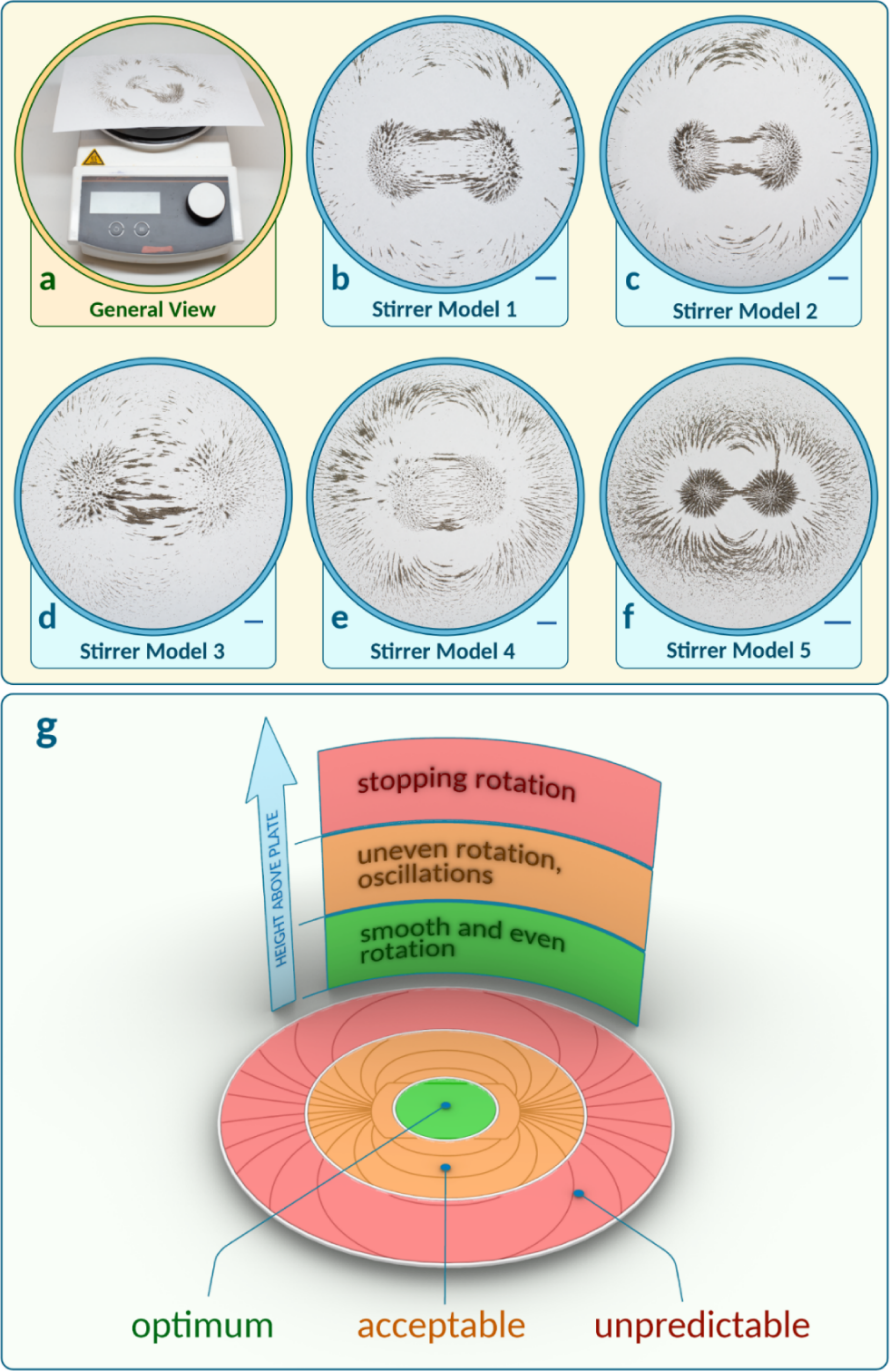
Visualization of the magnetic field on various stirrer models using magnetic nickel granules: (a) side view of the setup with a stirrer device; (b–f) top views of different stirrer devices with a scale bar corresponding to 1 cm; (g) an example scheme for optimizing stirring on the stirrer surface and along the central axis vertically.

With regard to the question of achieving better reproducibility for the reactions studied in the present article (Figures 6g and S11), stable reproducibility was observed in all of the studied processes (synthesis of nanoscale particles, catalyst preparation, and catalytic C–C bond formation reaction) when the reaction vessels were placed in a chosen “green” zone for a selected stirrer model (Figure 6b). For reproducibility in other synthetic laboratories, the final developed procedures should be performed in “green” stirring zones and properly described. In addition, we also suggest that manufacturers of stirrer devices should visually depict various stirring zones for particular stirrer models depending on the bar movement regime (see Supporting Information sections “Effect of geometrical position on the stirrer” and “Magnetic bar motion dynamics”; and other factors).

## Conclusions

This study underscores the important role that seemingly minor and previously overlooked experimental variations can play in the outcomes of chemical reactions, particularly in the context of utilizing magnetic stirrers. A primary observation was that the spatial positioning of the reaction vessels on a magnetic stirrer substantially influenced the stirring dynamics. This, in turn, affects the reaction kinetics and has a cascading effect, causing marked changes in results at both the microscale and nanoscale.

In particular, major differences were found in the size (2.0 ± 0.4 nm vs 1.3 ± 0.3 nm) and synthesis rate of the nanoparticles obtained simultaneously with the same stirrer. During the synthesis of nanosized catalysts, differences were observed for metal nanoparticles (with sizes ranging from 1.7 ± 0.8 nm to 4.2 ± 1.8 nm) and their synthesis rates, as well as differences in the resulting support morphologies. The height of the carbon material pillar varied significantly from 0.9 to 14 mm. In the case of catalytic cross-coupling reactions, conversion differences of more than 10% were observed among adjacent vials, with nearly 2-fold variations between nonadjacent positions

We discerned variances in the rotational behavior of the commercially available stirring bars when the systems were arranged differently on the stirrer. Such differences in stirring patterns can profoundly influence the reaction outcomes. Recognizing these disparities is crucial; it offers a foundation for broadening the parameters that define reaction procedures and promotes a comprehensive understanding of experimental dynamics. This observation is important, as it suggests a need for a more detailed methodological description of scientific procedures involving magnetic stirrers.

Highlighting the significance of these findings, it is critical to emphasize that the implications are far-reaching. For example, without consistent and standardized positioning on a magnetic stirrer, experimental results may show discrepancies, even when the same protocol is followed. Such inconsistencies can impede scientific progress, leading to potentially misinterpreted results and conclusions.

The broader relevance of our work is in its call for action from the scientific community. This emphasizes the necessity of characterizing and standardizing stirring equipment and related protocols. This includes more detailed aspects, such as the spatial arrangement of the reaction vessels on a magnetic stirrer, as well as the types and sizes of both the reaction vessels and the stirring bars. Disregarding these detailed parameters can introduce volatility, which can undermine the consistency of outcomes across different studies. We also recommend that a sufficient number of control experiments should be performed with one vessel in an unimpeded stirring area to obtain the final results.

Of course, different reactions may have different degrees of sensitivity to stirring; some reactions may not be sensitive noticeably, whereas other reactions may be highly sensitive. In particular, homogeneous systems are likely to be less sensitive than heterogeneous systems. However, these factors are rather difficult to assume a priori. Many initially homogeneous systems may de facto develop into microheterogeneous or colloidal states (i.e., “cocktail”-type catalysis). In photochemical systems, the light penetration depth may create local reaction zones where mixing is critically important to achieve reproducible reactivity and to reduce the bleaching of dyes. Importantly, temperature and viscosity gradients and transient concentration gradients due to the gradual introduction of reaction components may affect even completely homogeneous systems.

In essence, our research sheds light on an overlooked yet significant facet of experimental processes. Ensuring reproducibility and accuracy is fundamental in scientific endeavors, and this study underscores the importance of paying attention to even the minutiae of experimental setups to achieve that end.

## Supplementary Material









## Notes

Key factors of reproducibility, photos of the real and widespread reaction setup, experimental section, stirring comparison under different conditions, screening of various reactions, ejection of reagents, bar movements and stirring results, magnetic field visualization (PDF)
Video of real reaction setup 1 (MP4)
Video of real reaction setup 2 (MP4)
Video of real reaction setup 3 (MP4)
