# Supplementary material for: Magnetic Stirring May Cause Irreproducible Results in Chemical Reactions
Source: JACS Au. 2025 Jun 11;5(8):3789–98. doi: 10.1021/jacsau.5c00412 (PMC12381720; doi:10.1021/jacsau.5c00412)
Supplement: Supplementary file 1 [file au5c00412_si_001.pdf]

Supporting Information for the article

Magnetic Stirring May Cause Irreproducible Results in Chemical Reactions

Vera A. Cherepanova, Evgeniy G. Gordeev, Valentine P. Ananikov*

Zelinsky Institute of Organic Chemistry, Russian Academy of Sciences,

Leninsky prospekt 47, Moscow, 119991, Russia.

https://AnanikovLab.ru; Email: *val@ioc.ac.ru*

CONTENT

KEY FACTORS EFFECTING REPRODUCIBILITY S2

SOME REAL-LIFE EXAMPLES OF MAGNETIC STIRRER USAGE S3

REACTION SETUPS S7

EXPERIMENTAL S10

MAGNETIC BARS CHARACTERISTICS S15

COMPARISON OF STIRRING METHODS S16

EFFECT OF GEOMETRICAL POSITION ON THE STIRRER S20

MODELING OF THE INTERACTION BETWEEN MAGNETS BY FINITE ELEMENT METHOD S21

COMPARISON OF DIFFERENT STIR BAR SIZES AND SHAPES S25

MICROSCOPY STUDY OF Pd NANOPARTICLES DEPOSITED ON NANOTUBES S28

MICROSCOPY STUDY OF NANOTUBE CONGLOMERATES S29

REPRODUCIBILITY SCREENING IN DIFFERENT REACTIONS S30

EJECTION OF REAGENTS FROM THE REACTION SYSTEM DUE TO STIRRING S33

MAGNETIC BAR MOTION DYNAMICS S34

REACTIONS IN DIFFERENT VESSELS S44

VISUALIZATION OF THE MAGNETIC FIELD EFFECT S45

VISUALIZATION WITH SILICONE-BASED FERROFLUID S47

REFERENCES S47

KEY FACTORS EFFECTING REPRODUCIBILITY

Table S1. Main factors causing poor reproducibility in chemical research.[a]

Title	Description	Probability
Human Error	Simple mistakes like mismeasuring a reactant, confusing reagents, or misreading an instrument can lead to irreproducible results.	Very Often
Purity of Reactants	The purity of the starting materials plays a crucial role. Impurities, even in small amounts, can interfere with the desired reaction, leading to different products or yields.	Often
Reaction Conditions	Variations in temperature, pressure, or concentration can affect reaction outcomes. Some reactions are very sensitive to the presence of moisture (water) or oxygen.	Often
Solvent Effects	The choice of solvent can significantly influence reaction outcomes. Solvents can participate in reactions, stabilize certain intermediates, or even change the course of the reaction altogether.	Often
Instrumentation Variability	Different instruments or equipment might have slight variations in calibration or operation. For instance, a heating mantle from one manufacturer might heat slightly differently from another.	Often
Ambiguities in Experimental Descriptions	If a published procedure is not described in detail or lacks certain critical information, it can be challenging for others to reproduce the results faithfully.	Rare
Trace Impurities that Act as Catalysts	Even minute amounts of unintended substances in a reaction mixture can act as catalysts, accelerating or altering the course of the intended reaction. These impurities might arise from previous reactions, contamination of reagents, or even from lab equipment.	Rare
Complexity of the Reaction	Multi-step reactions with several intermediates can introduce multiple points of variability and potential failure.	Rare
Research Integrity	Instances where researchers might display dishonest behavior, fabricate data, or deliberately misrepresent results can significantly contribute to irreproducibility. Ethical conduct is fundamental to scientific progress and credibility.	Rare (hopefully)

[a] Assessing the factors by their likelihood of causing irreproducibility (the Probability column) can be subjective and dependent on the specific context of a laboratory or research environment. It is given here based on general experience and common issues faced in a typical lab setting and should be considered as an estimative tentative ranking.

SOME REAL-LIFE EXAMPLES OF MAGNETIC STIRRER USAGE

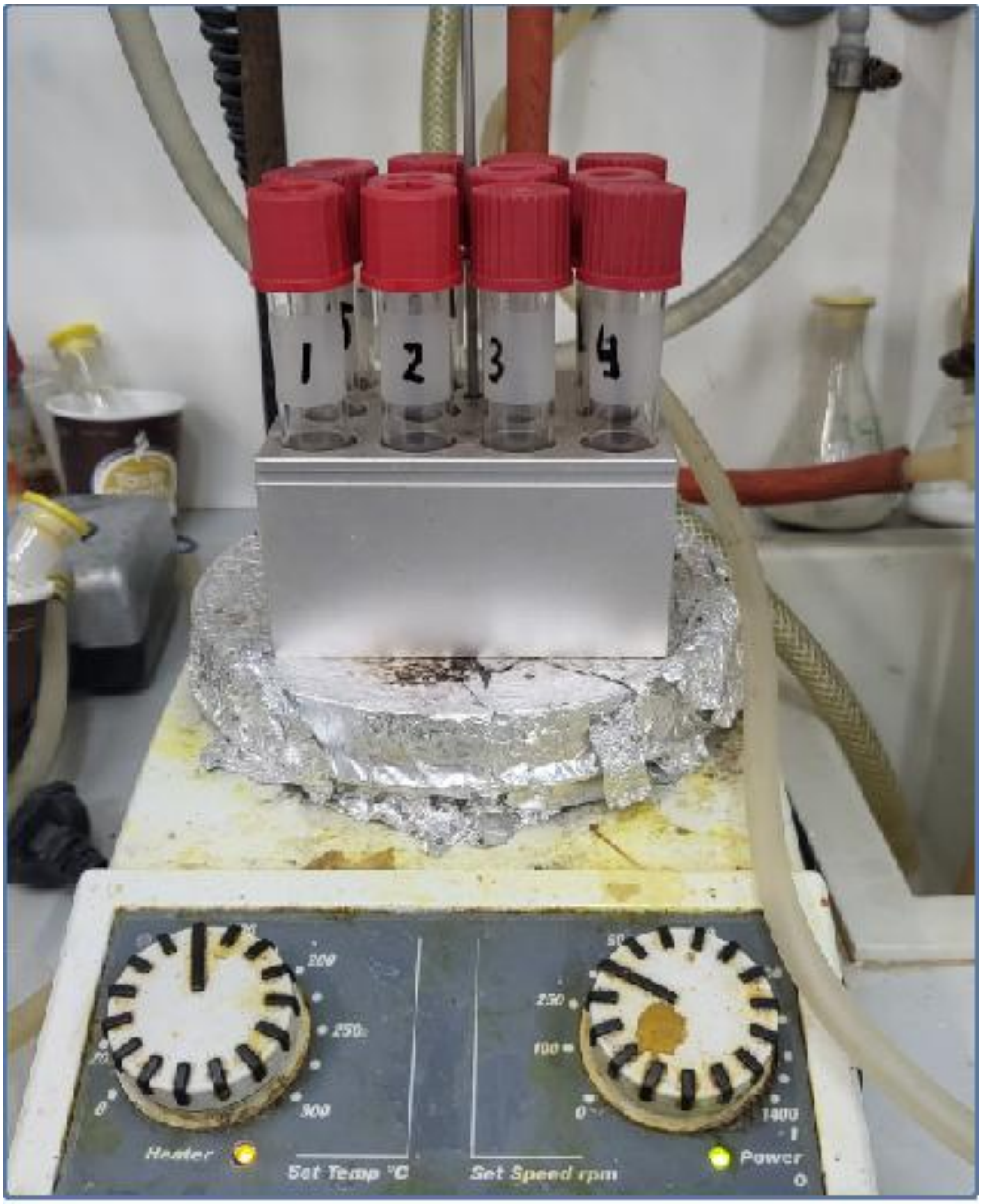

Figure S1. Typical setup for carrying out reactions simultaneously, with the stirring effect to be observed.

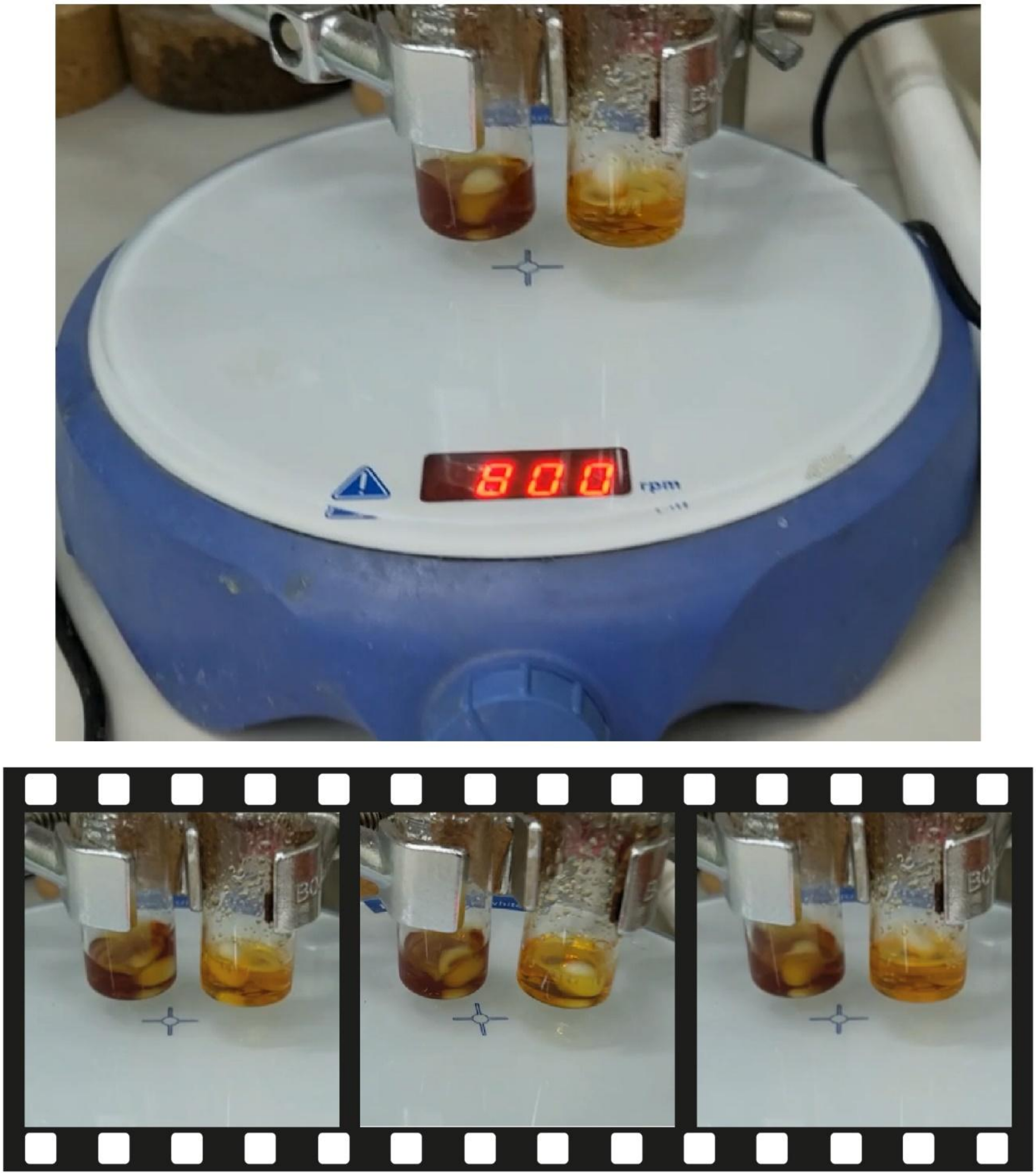

Figure S2. Typical setup of two reactions simultaneously. The vessels are at different distances from the center and at random heights. Visually, the bar movements are chaotic and do not agree with each other, probably mutually attracting, sometimes being outside the reaction liquid. The stirring effect is difficult to predict.

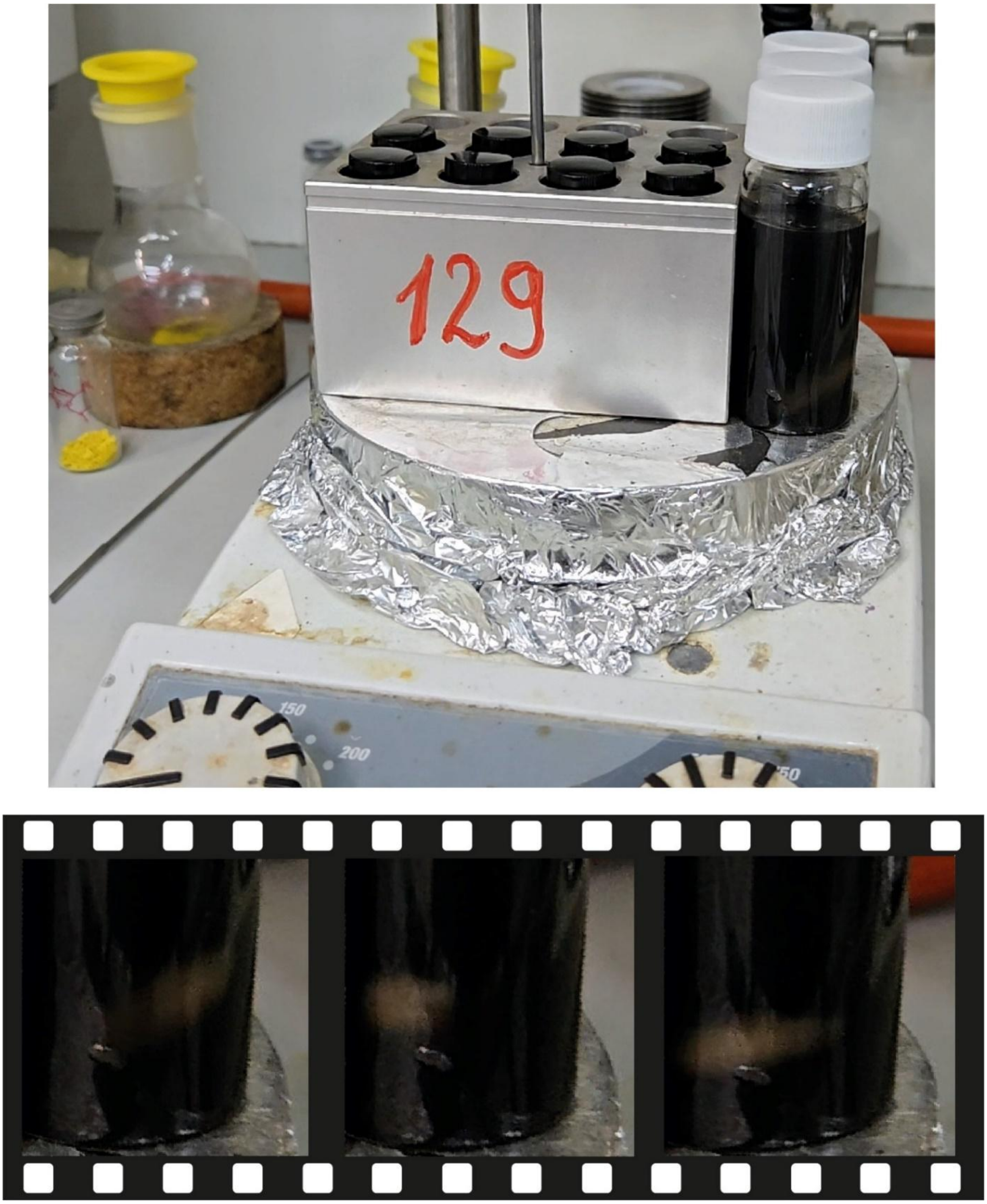

Figure S3. Setup of several reactions simultaneously, three of which occur in heterogeneous systems. The freeze-frame shots show the bar positions in one of them. The bar is above the bottom surface and tilts at an angle to the horizontal plane.

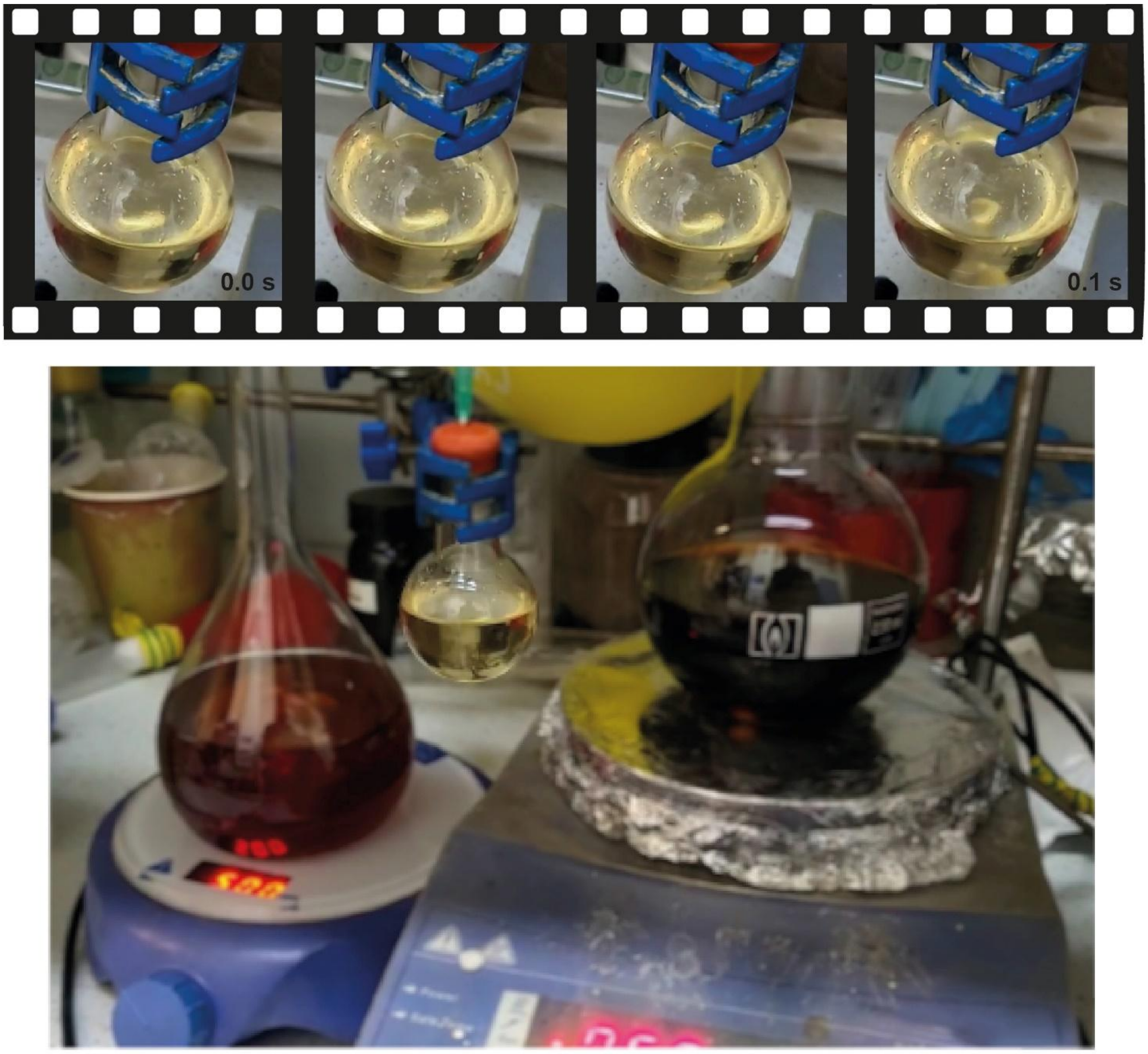

Figure S4. An extreme case of the reaction setup in a flask between two stirrers (a small flask in the middle between two large ones). The set stirring speeds are 500 rpm and 750 rpm, so the bar should make one turn in no more than 0.12 seconds (if take a lower speed). The freeze-frame shots show uneven movement, in 0.1 seconds the bar has turned by an angle of less than 180°.

REACTION SETUPS

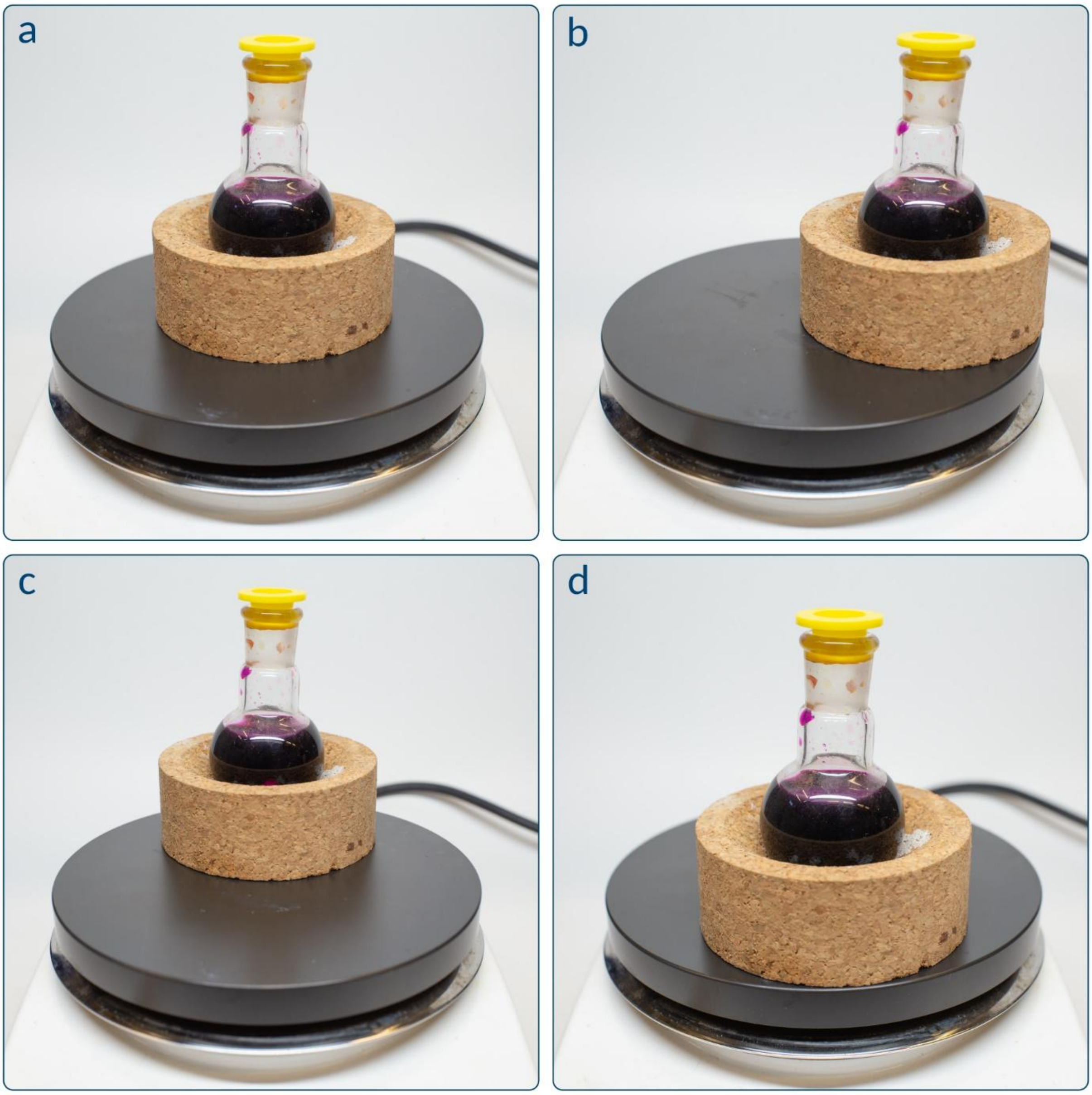

Figure S5. The flask in a cork stand on the stirrer heating surface: (a) – flask in the center, (b) – flask on the right edge, (c) – flask on the rear edge, (d) - flask on the front edge. Typically, little attention is given to positioning, and each of the locations may be used randomly among researchers.

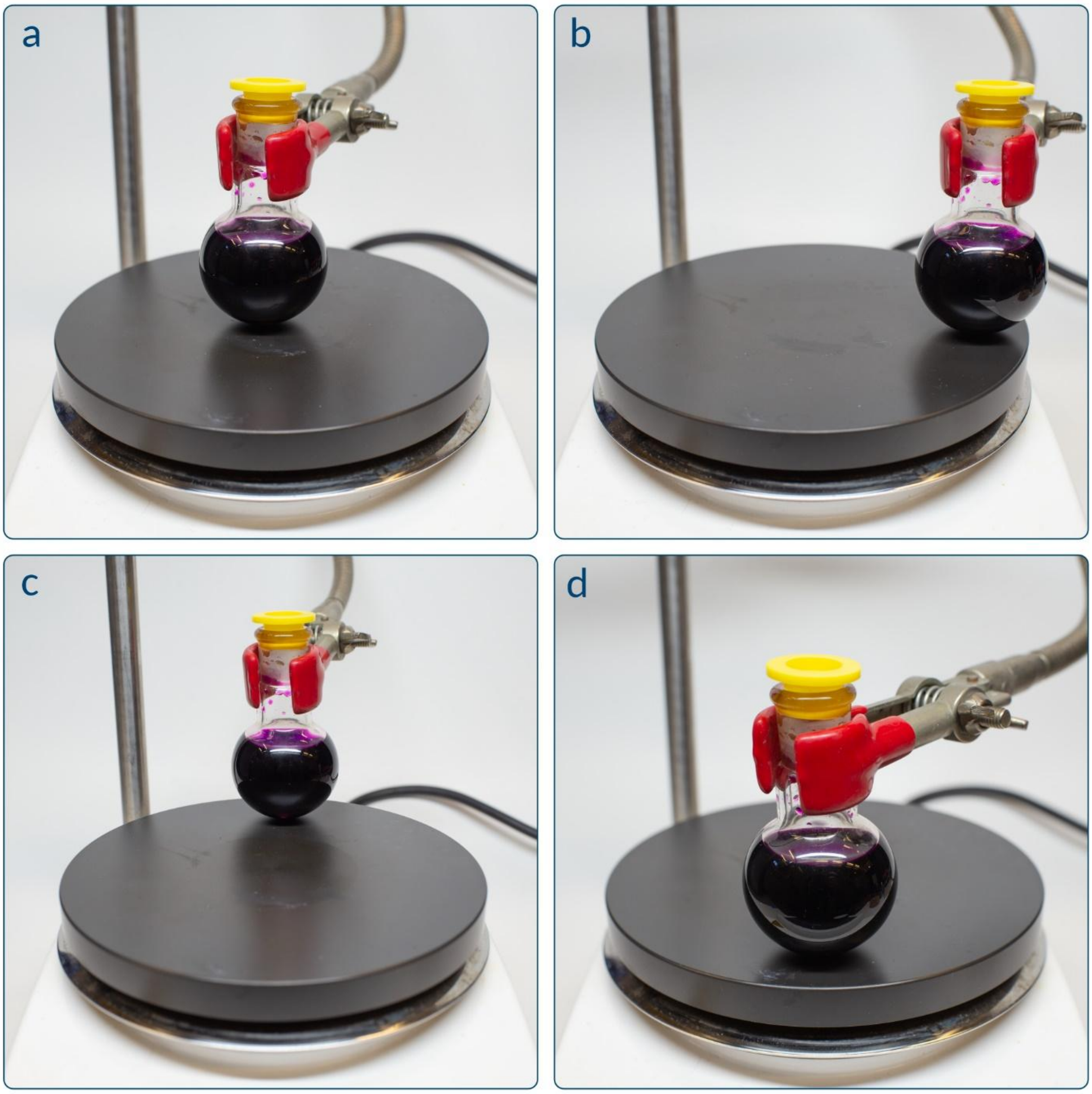

Figure S6. The flask in the clamp on the stirrer heating surface: (a) – flask in the center, (b) – flask on the right edge, (c) – flask on the rear edge, (d) - flask on the front edge. Typically, little attention is paid to positioning, and each of the locations may be used randomly among researchers.

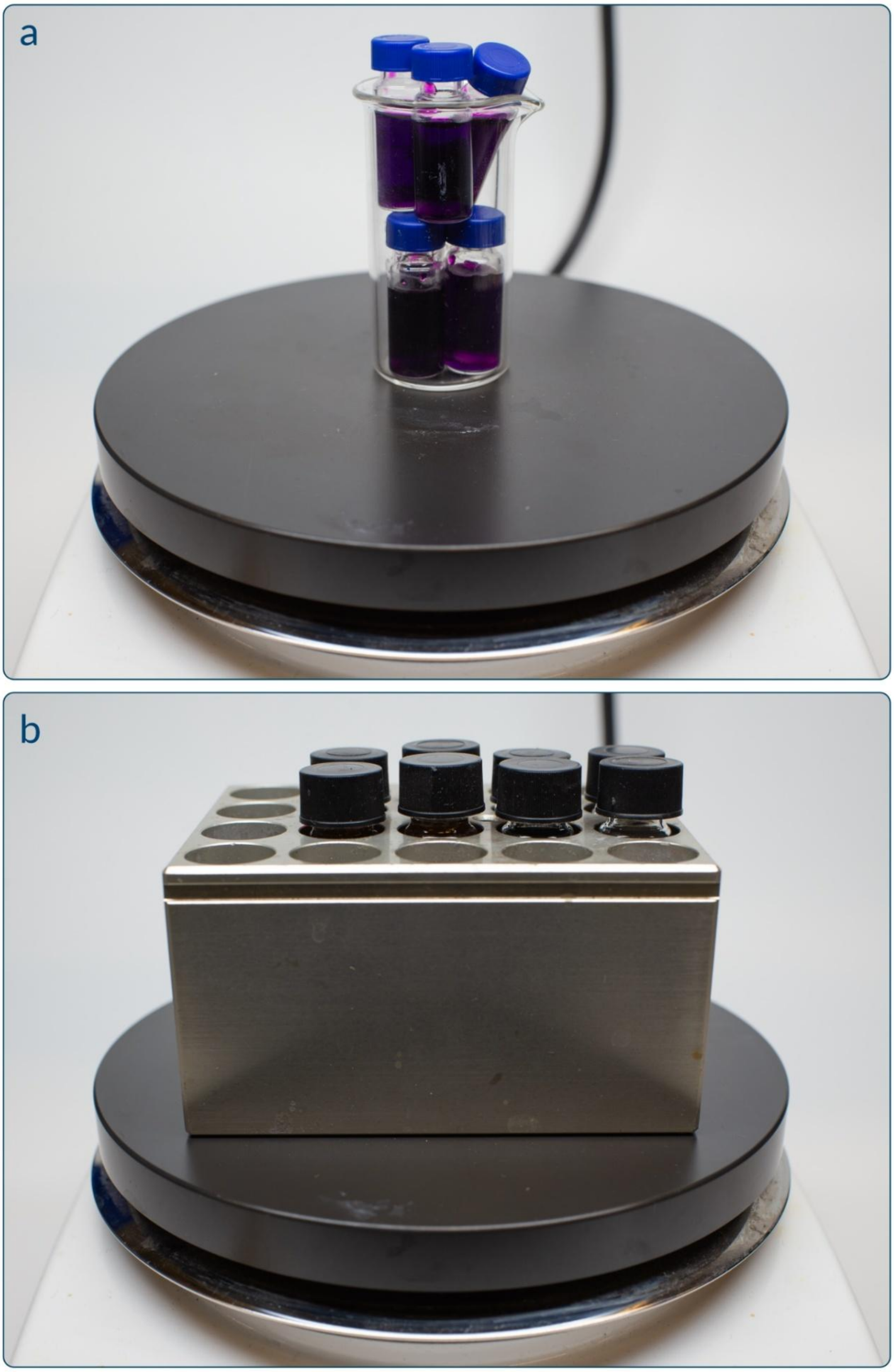

Figure S7. Staging multiple reactions simultaneously: (a) – vials in the glass, (b) – vials in the heating block. In practical experiments, very common mixing setups with unavoidable variations in the location of the stirrer.

EXPERIMENTAL

Magnetic stirrers used. Heidolph MR Hei-Tec, Corning® PC-420D, IKA® color squid white, IKA® RCT basic, IKA® big squid white, ULAB® US-1550A stirrer.

Materials used. Phenylboronic acid (Fluorochem Ltd., Hadfield, UK), potassium carbonate (Reachem, Moscow, Russia), laboratory-grade ethanol (Merck KGaA, Darmstadt, Germany), and palladium on carbon 1% (Sigma-Aldrich, St. Louis, MO, USA) were purchased from commercial sources.

Visual observation of differences

Stand for visual observation. A stand was developed for geometric quantification. The stand held the vials both horizontally and vertically in relation to the center of the stirrer surface. This design enables multiple reactions to be carried out in parallel at fixed positions. The stand was set along the diameter of the heating plate.

Pd/C catalyst preparation. $Pd_2dba_3 \cdot CHCl_3$ was synthesized according to the reported procedure.[1] $Pd_2dba_3 \cdot CHCl_3$ (7.5 mg) was dissolved in 60 ml of chloroform, and then 3.95 ml of solution and 20 mg of multiwalled carbon nanotubes were added to each of 15 thoroughly cleaned vials (Microlab Scientific, color clear, vial volume 4 ml, cap type: 13-425, 15 × 45 mm). The complex dissociation was carried out at room temperature using a Heidolph MR Hei-Tec stirrer at 500 rpm. A specially designed stand was installed on its surface (Figure 2b), consisting of 3 identical levels of 5 vial holders each, symmetrical with respect to the central axis. A new set of bars (VWR® PTFE cylindrical stirrer bar 8 × 3 mm) was placed in the vials and used during the first experiment, and these bars were used for all subsequent experiments. Complex decomposition was detected visually by a color change. The decomposition process was recorded using time-lapse photography with a Nikon D610 camera with a Nikon AF-S Nikkor 35 mm lens. Stirring was paused for at least 30 seconds before each photo was taken to allow the carbon material to settle, ensuring the color could be seen clearly.

Data processing. For the resulting images, the color of the solution was averaged and converted into numerical values. For this purpose, a consistent area was marked in all time-lapse photos within each vial, devoid of external interferences such as glare or carbon particles. The average solution color within this region was converted to HEX format and subsequently plotted in the xyY color space. This process produced 15 distinct graphs, each mapping the color change for a specific vial. Each of these graphs was fitted with a trend line, with quadratic trend lines demonstrating the best fit ($R^2 > 0.9$). The discoloration rate was calculated from the trend line's derivatives. For symmetrical positions, the graph with the highest R^2 value was prioritized.

Video recording of bar motion. In the stand described above, the vials were fixed alternately on one level. Each vial with stir bars was recorded individually for at least two minutes, including the acceleration time, using an Andonstar AD409-Pro tabletop microscope at 60 fps, and viewed through the vial's neck. Although the narrowness of the vial neck obscured the tiny outer rim of the vial bottom, the consistent shape of the bar allowed easy extrapolation. More than 7000 frames were taken by the camera in approximately two minutes. For visual comparison, we selected 250 frames of stationary stirring (i.e., omitting the bar acceleration time). Using graphic editor software, these images were superimposed, and the area within which the bar was located during the stirring process was determined.

For a comprehensive visual understanding, every 100 frames were overlaid to characterize the stirring mechanism.

Video recording of changes in bar motion when the vessel was shifted along the horizontal axis. A radial line was drawn on the surface parallel to the camera movement. The vessel with the bar was shifted synchronously with the camera movement.

Video recording of changes in bar motion when the vessel was shifted along the vertical axis. Identical foam board squares, each 5.5 mm thick, were placed on the stirrer surface. A glass surface with a magnetic bar (VWR® PTFE cylindrical stirrer bar 20 × 6 mm) was placed on a stack of squares. At each level, the rotation speed was checked with a tachometer (CEM AT-6), and the movement nature was recorded by video (Nikon D610 camera) for two minutes.

Decomposition of the $Pd_2dba_3·CHCl_3$ complex. 15 mg of $Pd_2dba_3·CHCl_3$ was dissolved in 60 ml of $CHCl_3$, and then 3.95 ml of solution was added to each thoroughly cleaned vial (Microlab Scientific, color clear, vial volume 4 ml, cap type: 13-425, 15 × 45 mm). Complex decomposition was carried out at room temperature using a Heidolph MR Hei-Tec stirrer at 500 rpm. A specially designed stand was installed on its surface (Figure 2b), consisting of 3 identical levels of 5 vial holders each, symmetrical with respect to the central axis. A new set of bars (VWR® PTFE cylindrical stirrer bar 8 × 3 mm) was placed in the vials and used during the first experiment, and these bars were used for all subsequent experiments. Complex decomposition was detected visually by a color change. The decomposition process was recorded using time-lapse photography with a Nikon D610 camera with a Nikon AF-S Nikkor 35 mm lens. Photomonitoring was simplified without suspended particles in the mixture, eliminating the need to let them settle before each snapshot.

TEM measurements. A targeted approach was used to optimize the analytical measurements.[2] During the reaction, an aliquot of 0.5 ml was taken from the solution with continuous stirring. Prior to measurement, the test solution, diluted in isopropanol, was applied to a 3 mm copper grid with a carbon film and fixed in a grid holder. The morphology of the samples was

examined using a Hitachi HT 7700 transmission electron microscope (TEM). Images were taken in bright field TEM mode at an accelerating voltage of 100 kV.

NMR monitoring

NMR monitoring of the Pd/C catalyst preparation. A 4 ml solution of $Pd_2dba_3{\cdot}CHCl_3$ in $CDCl_3$ (2 $mg{\cdot}ml^{-1}$) was put in a vial containing 20 mg of MWCNTs. The vials were placed on a three-tier stand. The reaction was carried out at room temperature using a Heidolph MR Hei-Tec stirrer at 500 rpm. During the imaging process, 0.1 ml aliquots of the reaction mixture and 0.5 ml of pure $CDCl_3$ were placed in NMR tubes at specified time intervals. The carbon material was removed from the solution by settling. NMR analysis was performed immediately after sample preparation by NMR spectroscopy (Bruker Avance II 600 spectrometer Bruker Corporation, Billerica, MA, USA). Immediately after the analysis, the entire volume of the solution was returned to the reaction mixture. The concentrations of Pd_2dba_3 and free dba were calculated according to a previously reported method.[1]

NMR monitoring of the reaction leading to the formation of Pd nanoparticles. A 4 ml solution of $Pd_2dba_3{\cdot}CHCl_3$ in $CDCl_3$ (2 $mg{\cdot}ml^{-1}$) was put in the same vial. The vials were placed on a three-tier stand. The reaction was carried out at room temperature using a Heidolph MR Hei-Tec stirrer at 500 rpm. During the imaging process, 0.1 ml aliquots of the reaction mixture and 0.5 ml of pure $CDCl_3$ were placed in NMR tubes at specified time intervals. NMR analysis was performed immediately after sample preparation by NMR spectroscopy (Bruker Avance II 600 spectrometer Bruker Corporation, Billerica, MA, USA). Right after the analysis, the entire volume of the solution was returned to the reaction mixture. The concentrations of Pd_2dba_3 and free dba were calculated according to a previously reported method.[1]

Study of catalytic systems

Setup for catalytic systems. The setup consists of a central vial, four vials at an intermediate distance from the stirrer's center, and four at the farthest distance. With this configuration, each experiment yielded results for three distinct positions.

Suzuki-Miyaura reaction. 4-Iodoaniline (119.7 mg), boronic acid (109.6 mg), and potassium carbonate (111.6 mg) were dissolved in 30 ml of ethanol/water mixture (5:1). A sample of commercial Pd/C catalyst (10.6 mg), a magnetic bar and a volume of solution (2.9 ml) were put in each of nine vials. The vials were placed in a specially designed stand (Figure 5b), which allowed nine vials to be placed on the surface of the stirrer. Four vials were positioned so that the circumference of the vial and the circumference of the tile were in internal contact, four other vials

touched a much smaller circle, and one vial was in the center. All vials were placed at the minimum possible height from the stirrer surface, which was limited by the thickness of the water bath glass.

The selection of phenylboronic acid and iodoaniline as substrates was guided by the convenience of analyzing the spectrum of such a reaction mixture. Additionally, the chosen reaction proceeded efficiently within an hour in a water bath at 70 °C. The reaction was carried out for one hour at 250 rpm and 70 °C, with uniform heating provided by two mechanical stirrers, IKA Overhead Stirrer RW11, submerged in water and placed symmetrically between the vials at the edge of the plate. At the end of the reaction, a sample (500 µl) of the Suzuki-Miyaura reaction mixture was taken, and a standard (*p*-dinitrobenzene) dissolved in DMSO-d^6 (100 µl) was added. The conversion was determined by NMR spectroscopy (Bruker Fourier 300 HD spectrometer, Bruker Corporation, Billerica, MA, USA). Data analysis was performed once three consistent outcomes were achieved for each position.

Equipment description

Bars description. 50 bars were weighed, and their length was measured. The measurement results were statistically analyzed. The density was measured for each bar material separately. The magnetic element and the shell were measured separately, and the volume was calculated geometrically on the basis of the 3D model.

Study of cracks in vials. Three new vials (Microlab Scientific, color clear, vial volume 4 ml, cap type: 13-425, 15 × 45 mm) were taken. The first one served as the standard. A solution of $Pd_2dba_3 \cdot CHCl_3$ in $CHCl_3$ (0.25 mg·ml^{-1}) and a new stir bar were added to the second and third vials, and nanotubes were added to the third vial. The second and third vials were mixed continuously for 35 hours at 500 rpm at off-center positions. After completion, the vials were rinsed with acetone and dried. Macro photography was carried out using a Nikon D610 camera with a Nikon AF-S Nikkor 35 mm lens.

For electron microscopic imaging, sections were cut from the vials, mounted on a 25 mm aluminum sample stub and fixed with Leit-C-Plast. A targeted approach was used to optimize the analytical measurements.[2] A thin film of carbon (20 nm) was deposited *via* a previously described method.[3] Observations were made *via* a Hitachi Regulus 8230 field emission scanning electron microscope (FE-SEM). Images were taken in secondary electron mode at an accelerating voltage of 10 kV and at a working distance of 10.0–11.2 mm. The morphology of the samples was studied considering the possible influence of the carbon coating on the surface.[3]

Reactions without stirring. As a control experiment, we have carried out the experiments without stirring and the reactions expectedly proceeded significantly slower.

Visualization of magnetic fields. An equal amount of small nickel granules was placed on the surfaces of magnetic stirrers without actually stirring. Photographs were taken using a Nikon D610 camera with a Nikon AF-S Nikkor 35 mm lens.

Measurements of magnetic field changes with the addition of a vial or a vial with a bar were made in a similar manner. Granules were added to the magnetic stirrer surface with an empty vial placed on them, and then a bar was added to the vial.

Figure 6b in the article text corresponds to the Heidolph MR Hei-Tec stirrer, Figure 6c to IKA® RCT basic, Figure 6d to ULAB® US-1550A, Figure 6e to Corning® PC-420D and Figure 6f to IKA® color squid white.

For imaging with ferrofluid (18% iron oxide, 62% silicone), it was poured into a glass vessel with a diameter slightly larger than the stirrer surface. For imaging with a bar (VWR® PTFE cylindrical stirrer bar 8 × 3 mm), it was added to the same system without changing the fluid volume. The imaging was performed using a Nikon D610 camera.

Computational Details. Modeling of the interaction between the stirrer under-plate magnet and the stir bar was performed using the FEMM 4.2 software package.[4] The problem was formulated in two dimensions. Open boundary conditions (Dirichlet edge type and 7 layers) were used for calculations. The environment for the magnets was air. The width of the stirrer under-plate magnet was 50 mm, and its height was 5 mm. The dimensions of the stir bar are as follows: width, 5.5 mm (X-axis dimension); height, 1.6 mm (Y-axis dimension). The vectors of magnetization of both magnets are parallel to the X-axis and have opposite directions. The coercivity of the material of both magnets was $16 \cdot 10^4$ $A \cdot m^{-1}$. The finite element mesh contained 7500 – 12100 nodes (the number of mesh nodes varied depending on the studied model).

MAGNETIC BARS CHARACTERISTICS

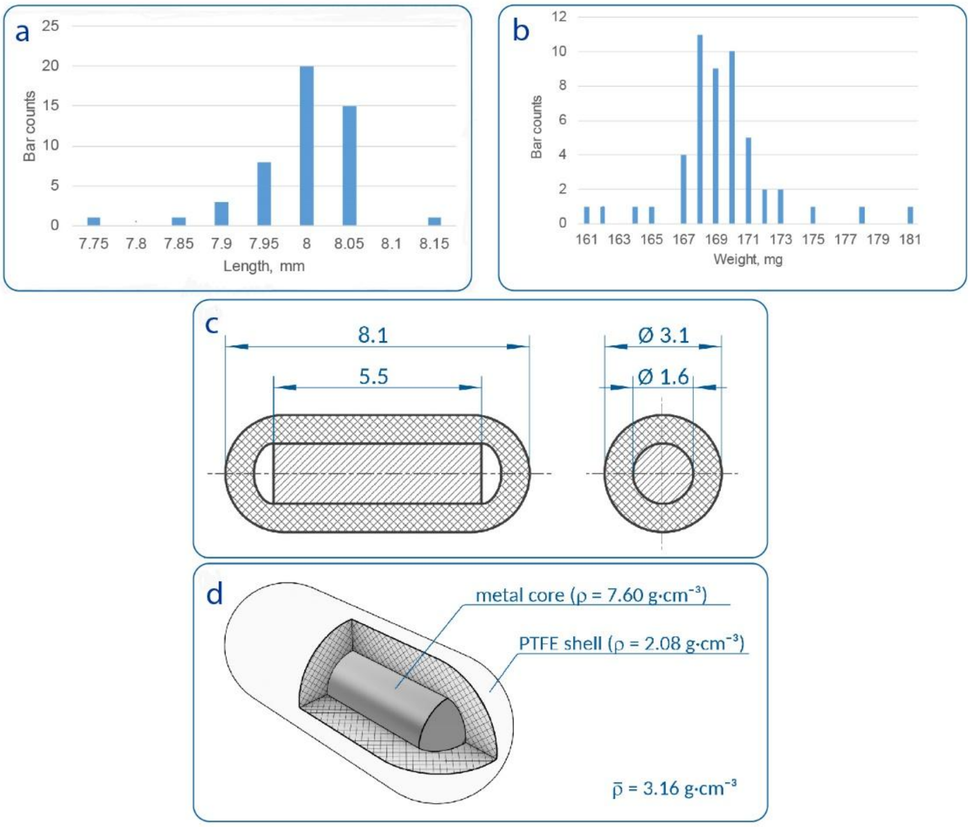

Figure S8. Bars characteristics. Statistics: (a) – length distribution, (b) – weight distribution. Description of a specific bar: (c) – linear dimensions, (d) – bar element density and average bar density.

COMPARISON OF STIRRING METHODS

Magnetic and mechanical stirrers

$Pd_2dba_3{\cdot}CHCl_3$ (16 mg) was dissolved in 8 ml of $CDCl_3$ and separated into two 4 ml vials. 40 mg of nanotubes and the appropriate bar were added to each vial. A VWR® PTFE cylindrical stirrer bar 8 × 3 mm was used for the systems. In a mechanically stirred system, the bar was held on by a specifically designed holder that attached it to the rotor. The solutions were stirred for 5 hours. During the reaction, 0.1 ml aliquots of the reaction mixture and 0.5 ml of pure $CDCl_3$ were places in NMR tubes at specified time intervals. NMR analysis was performed immediately after sample preparation. The reaction progress was determined by NMR monitoring.

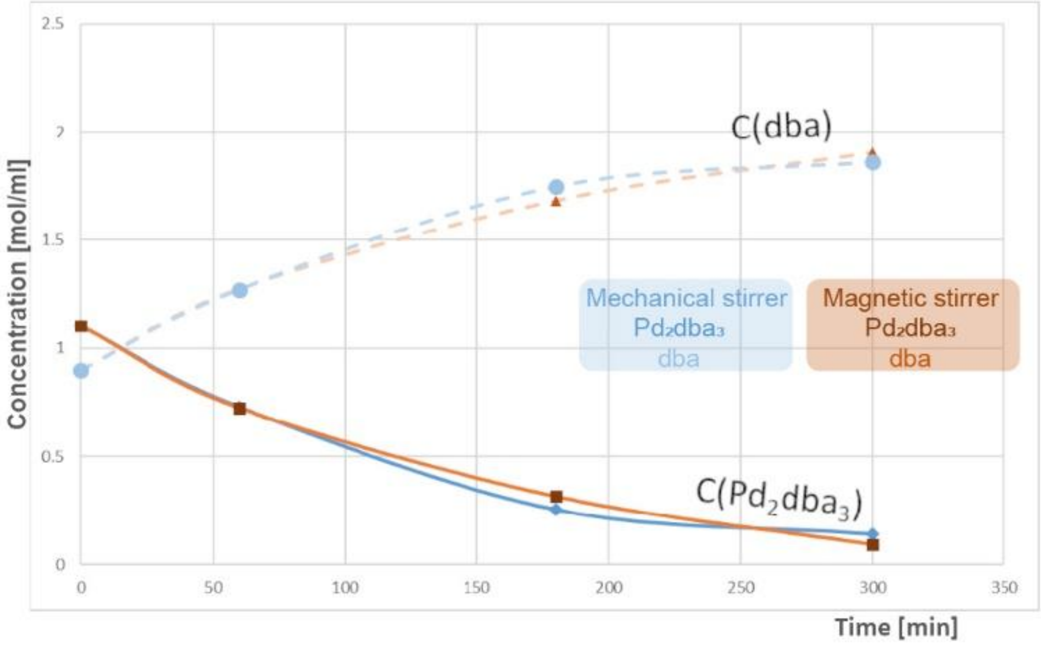

Figure S9. ^{1}H NMR monitoring of the reaction: the blue solid and dotted lines represent the Pd_2dba_3 and free dba concentrations, respectively, for the mechanical stirrer system, whereas the orange solid and dotted lines denote the same parameters for the magnetic stirrer system.

Experiment with a shaker device

$Pd_2dba_3{\cdot}CHCl_3$ (8 mg) was dissolved in 8 ml of $CDCl_3$ and separated into two 4 ml vials. 20 mg of nanotubes and the appropriate bar were added to each vial. The vials were placed on an OS-20 BioSan orbital shaker on an UP-12 shaker universal platform, one vial being in the center and another at the edge. The solutions were stirred for 2 hours. In the end 0.5 ml aliquots were sampled in NMR tubes, with 0.1 ml of pure $CDCl_3$ added. NMR analysis was performed immediately. The reaction progress was determined by NMR.

As a result, after two hours the reaction of Pd deposition on nanotubes proceeded in the same way – the complex concentration decreased from 0.59 mg·ml^{-1} to 0.37 mg·ml^{-1} in the center and 0.38 mg·ml^{-1} at the edge.

Factors changing in systems with different locations

The setup shown in Figure 3 allows performing 15 simultaneous reactions at various positions relative to the magnet of the magnetic stirrer. Initially, all vials contained a magenta-colored solution and an equal amount of carbon material. By the end, the vials display a colorless solution (due to the deposition of Pd on the carbon surface) with scattered carbon particles, resulting in a gray appearance and varying heights of the carbon material.

To standardize the synthesis of the Pd/C catalyst and subsequent reactions, the following parameters were analyzed:

● vial position relative to the center of the magnetic stirrer: defined by the horizontal and vertical axes. The number indicates the elevation of the system position, and the letter indicates its distance from the center. The coordinates directly influence bar rotation because of the varying magnetic field strength across different vial positions on the stirrer.

● discoloration rate: indirectly signifies the complex decomposition rate, shedding light on the distinct reaction kinetics across the systems.

● height of the nanotube pillar: In systems containing equal amounts of nanotubes, the height of the nanotube pillar varies (due to morphology and dispersion/density changes). The distinct inclinations of the bars during stirring caused some of them to collide constantly with the vial walls, leading to the breakdown of the nanotube clusters trapped between the bar and the wall. These fragmented conglomerates settled more loosely than did larger intact conglomerates.

● bar positions in the vial: The bar's movement is intrinsically tied to its location on the stirrer. The bars in vials away from the stirrer center tend to collide with the vial walls. If not fixed, the vial gradually drifts toward the stirrer's center, as the bar realigns itself horizontally. When fixed, the bar tilts to align with the magnetic field lines of the rotating stirrer. Variations in bar positioning are primarily responsible for differences in system parameters.

● nanoparticle sizes: Indeed, the palladium deposition rate on carbon materials influences the nanoparticle size. Given the variations in bar movement and consequently diverse reaction rates, differences in nanoparticle sizes are anticipated during both catalyst synthesis and in-solution nanoparticle formation.

Each of the system characteristics was systematically measured. For symmetrical placements, values were averaged, yet general trends remained consistent for both symmetrical setups and distinct experiments (Figure S10).

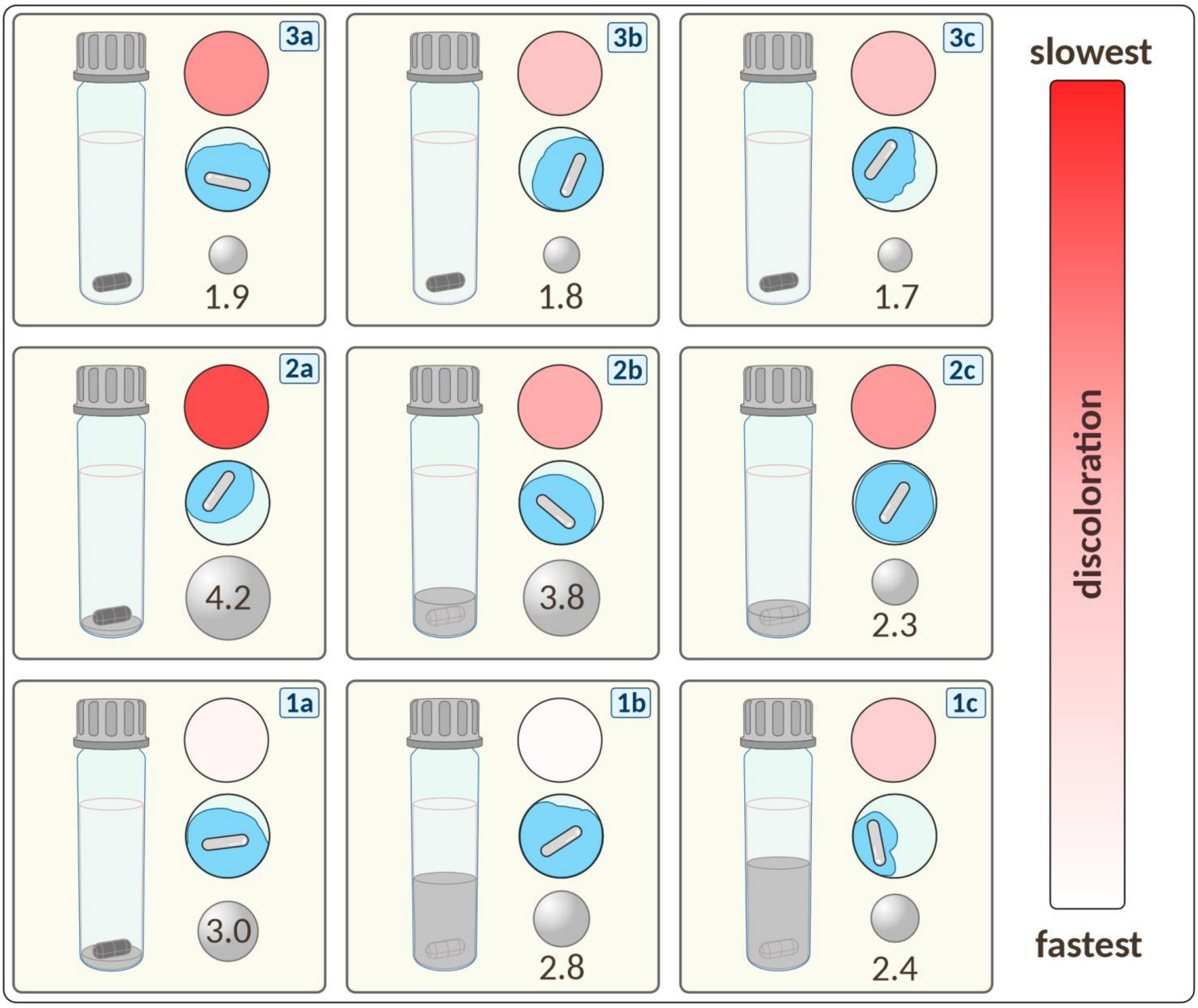

Figure S10. Overview of Pd/C synthesis systems on a stand. The central position corresponds to designation 1a. The numbering increases from bottom to top, and the letters follow from the center to the edge of the stirrer surface. The discoloration rate is visually represented by a color gradient: systems discoloring the quickest are white, whereas those discoloring the slowest appear red. The accompanying table details parameters such as the discoloration rate, nanotube pillar height, nanoparticle size, and overall bar position within each vial.

The most rapid discoloration occurred at the 1b position. The central 1a position followed shortly after. The 1c positions discolored considerably later. The earliest discoloration observed in the lower-level vials, distinctly different from other positions, was anticipated since the direct contact of the reaction vessel with the stirrer's surface is its standard mode of operation, a configuration optimized by the manufacturer. However, the subsequent discoloration sequence is less intuitive. Positions 3b and 3c demonstrated identical discoloration rates. Positions 2b and 2c followed in succession, with a less pronounced difference in their discoloration rates than their

lower-tier counterparts. The systems aligned with the central axis, 3a, followed by 2a, were the last ones to discolor.

The size of the resulting particles deposited on the nanotubes was correlated to some extent with the discoloration rate. The largest particle size was observed in the system with the longest discoloration time. Notably, although the top-tier systems varied in their rates, the particle sizes remained consistently similar.

Differences in the nanotube arrangement in the reaction mixtures within the vials were evident even to the naked eye. In certain systems, the nanotubes clung to the bar, whereas in others, they amassed to form a pillar, thereby appearing to occupy more volume. The most prominent nanotube pillar was discerned at the 1c position, with a slightly smaller one at 1b. A different trend was noticeable for the second tier: the 2b system manifested a slightly greater volume of nanotubes compared to 2c. For the centrally located systems on the first and second levels, the pillar height was notably reduced. Conversely, the top level displayed a distinct pattern: the nanotubes clung to the bars, not covering the entirety of the bottom surface. The observed pillar heights might be related to the varying bar movements and the subsequent grinding of the nanotube coils, resulting in a more dispersed arrangement. TEM analyses further confirmed this observation regarding the sizes of the nanotube conglomerates in the lower tier (Figure S20).

Another factor potentially contributing to the observed differences between systems is the area that the magnetic bar occupies during the stirring process (for more detailed illustrations of this factor see Figure S10 and Tables S3–S11).

EFFECT OF GEOMETRICAL POSITION ON THE STIRRER

Two stirrers were selected to observe the change in bar rotation: IKA® big squid white and Corning® PC-420D, with two bars: a VWR PTFE cylindrical stirrer bar of 25 × 6 mm and a VWR PTFE micro stirrer bar of 10 × 3 mm. Stirring was carried out in a 25 ml beaker with an inner diameter of 32 mm.

A ruler was attached to the surface of the stirrer to monitor the movement of the vessel and the bar. The vessel was moved simultaneously with the camera. The effective mixing zones were determined from the video frames. In the green zone, mixing is most effective. The boundary of this zone was determined by the position where the circle described by the bar no longer touched the center of the vessel. In the yellow zone, stirring is less effective. The boundary of this zone lies where the bar starts colliding with the vessel wall in its motion. In the red zone, stirring is the worst throughout the entire stirrer surface because the bar movement becomes erratic, as the bar degrees of freedom are limited by being pressed to the vessel wall.

Experiments have shown that the radius of the green zone is equal to half the bar length and that the radius of the yellow zone is inversely related to the bar size, but the relationship is not direct.

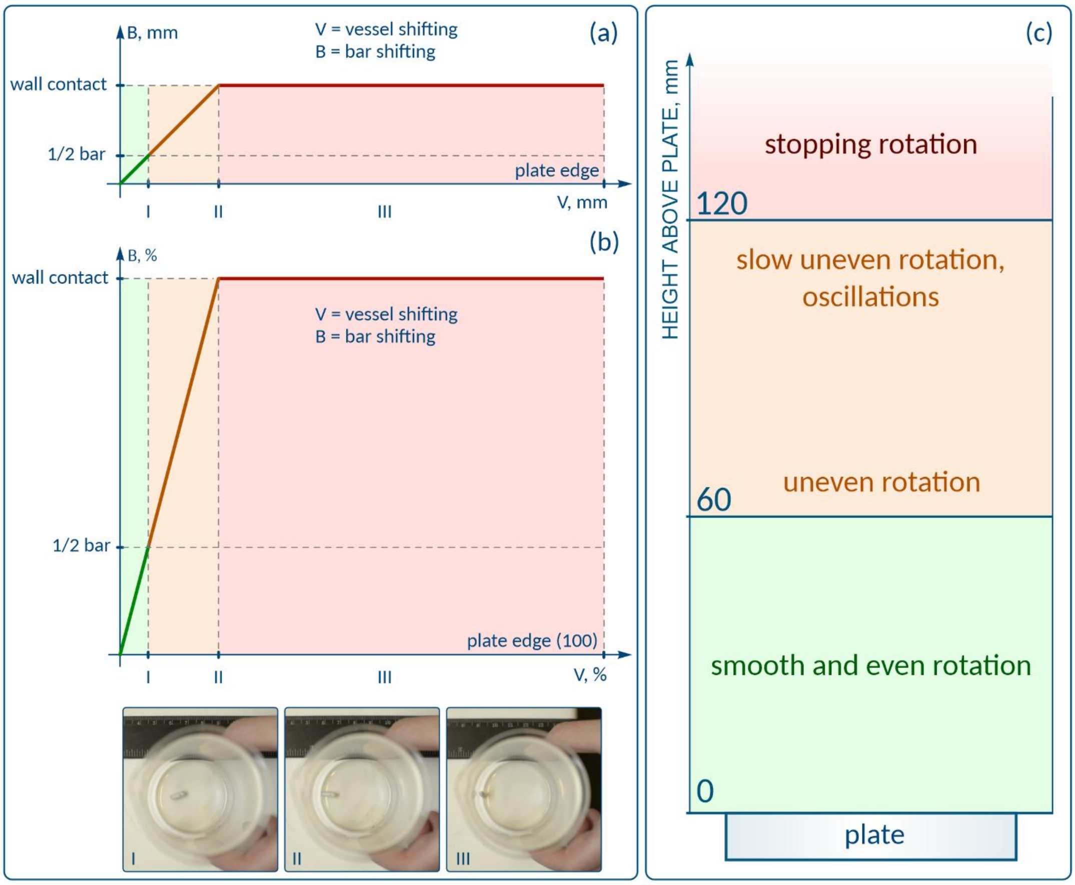

Figure S11. Graphs showing the results of the stirring zone analysis. Two-dimensional dependence of the bar offset relative to the vessel as the vessel is shifted from the stirrer center horizontally:

(a) – in millimeters, (b) – in percent; (c) – dependence of the bar rotation change with the vertical distance from the stirrer center.

MODELING OF THE INTERACTION BETWEEN MAGNETS BY FINITE ELEMENT METHOD

The magnetic field configuration for the magnet used in the magnetic stirrer was modeled using the finite element method (Figures S12). It is shown that the magnetic lines as well as the maps of magnetic induction B and field intensity H agree well with the shape of the magnetic fluid topography used to study the magnetic field of the stirrer (Figure S29). The magnetic field intensity gradually decreases from the center of the stirrer to its edges.

To theoretical study of the interaction between the stirrer under-plate magnet and the stir bar magnet, the two-magnet system was modeled using the finite element method (Figures S13, S14). As a result of the modeling, the values of the forces acting on the stir bar magnet were obtained when the stir bar magnet was gradually displaced relative to the stirrer under-plate magnet. The displacement of the stir bar magnet to the edge of the stirrer under-plate magnet results in an asymmetric action of the magnetic field on the stir bar magnet, which leads to the displacement of the stir bar from the center of the reaction vessel and unstable rotation of the stir bar (Figure S13b). As expected, moving the stir bar magnet away from the stirrer under-plate magnet leads to a gradual decrease in the interaction force between the magnets, which is particularly noticeable when the stir bar magnet is moved along the Y axis (Figure S14): the force component F_y decreases from -0.073 N to -0.0009 N (the negative force value indicates the attraction between the magnets in this direction).

The whole reaction system also contained a glass vial with some volume of chloroform. Since the glass, chloroform, and aluminum alloys of the stirrer plate are characterized by a relative magnetic permeability close to 1, their influence on the character of the field intensity distribution is negligible (Figure S15).

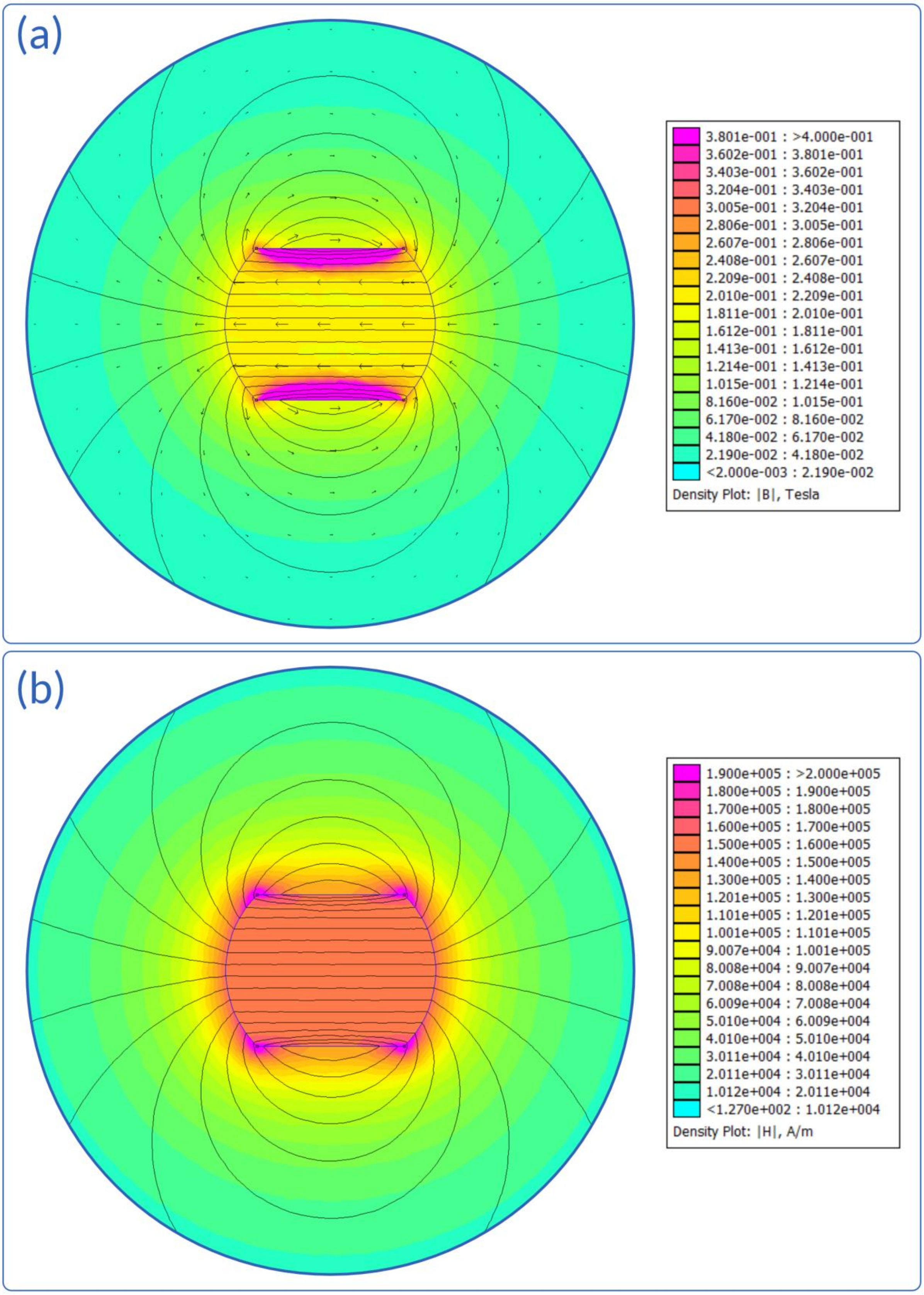

Figure S12. Maps of the flux density B (Tesla) (a) and field intensity H ($A \cdot m^{-1}$) calculated by the finite element method.

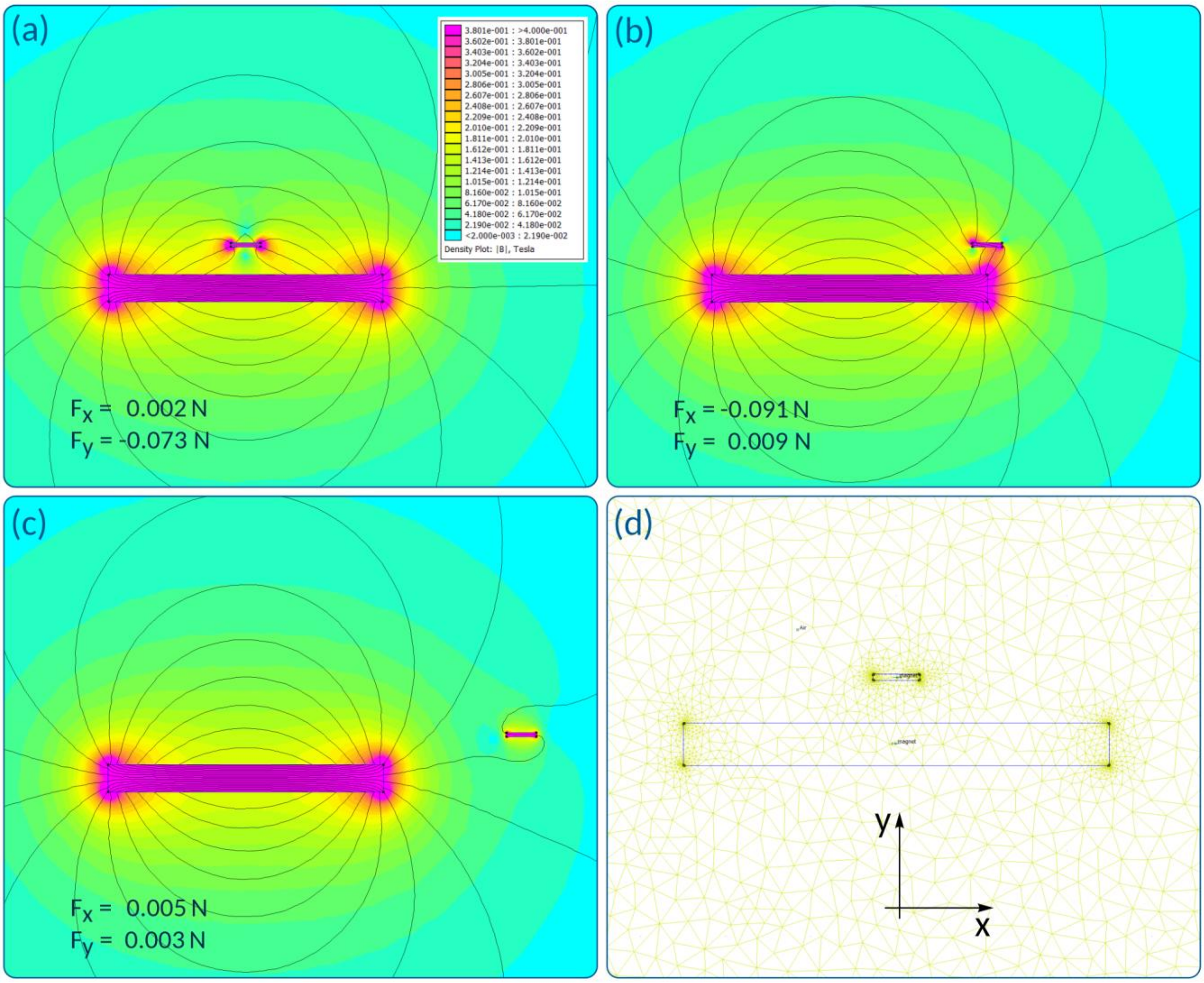

Figure S13. Magnetic field lines and maps of the B values (Tesla) for the system consisting of the stirrer under-plate magnet and stir bar at different positions of the two magnets: (a) the stir bar magnet is above the center of the stirrer under-plate magnet; (b) the stir bar magnet is displaced to the edge of the stirrer under-plate magnet; (c) the stir bar magnet is outside the geometric dimensions of the stirrer under-plate magnet; (d) finite element mesh used for modeling (shown for case (a) only). For each magnet arrangement, the X and Y forces (N) acting on the stir bar magnet are given.

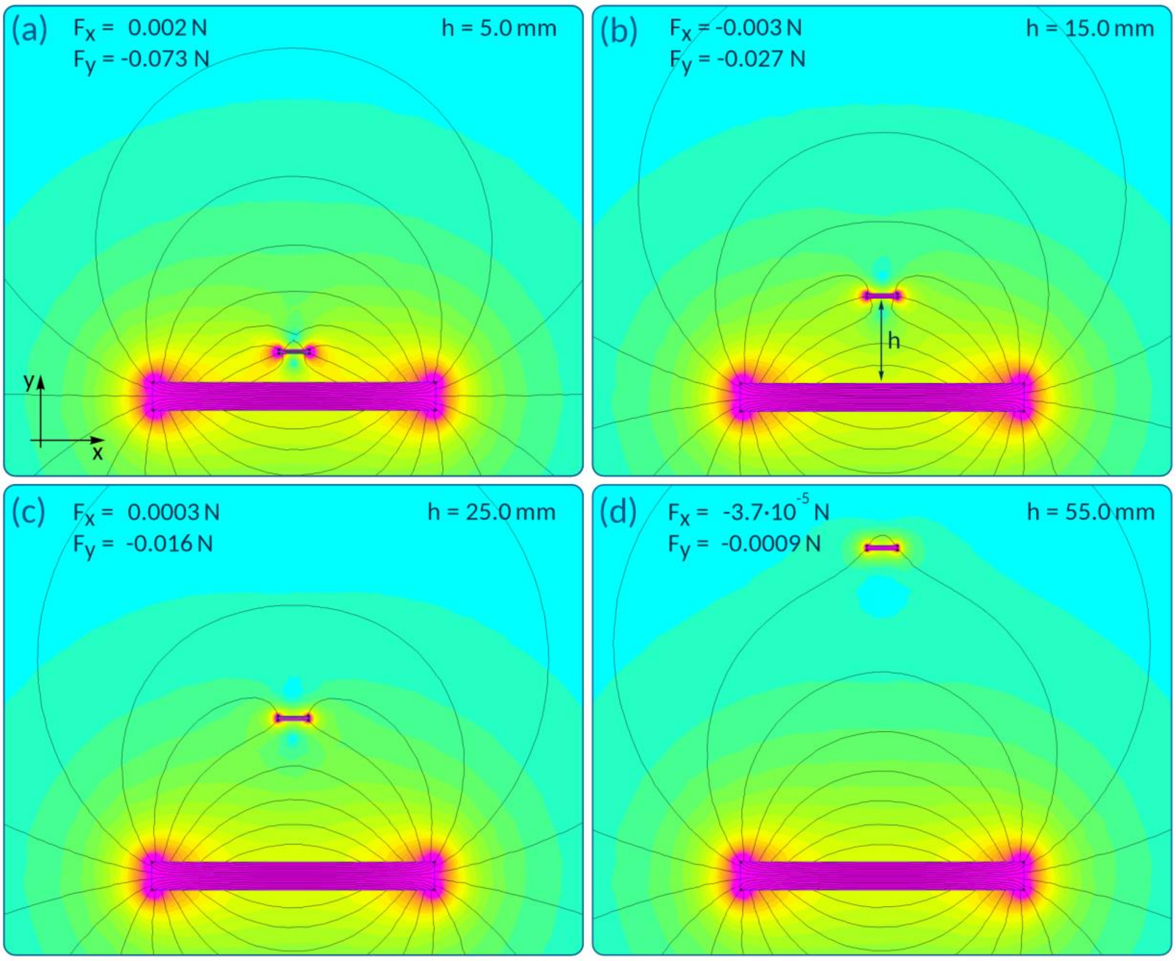

Figure S14. Magnetic field lines and maps of the B values (Tesla) for the system consisting of the stirrer under-plate magnet and stir bar at different distances (h, mm) above the center of under-plate magnet: (a) distance between magnets is 5 mm; (b) distance between magnets is 15 mm; (c) distance between magnets is 25 mm; (d) distance between magnets is 55 mm. For each distance, the X and Y forces (N) acting on the stir bar magnet are given.

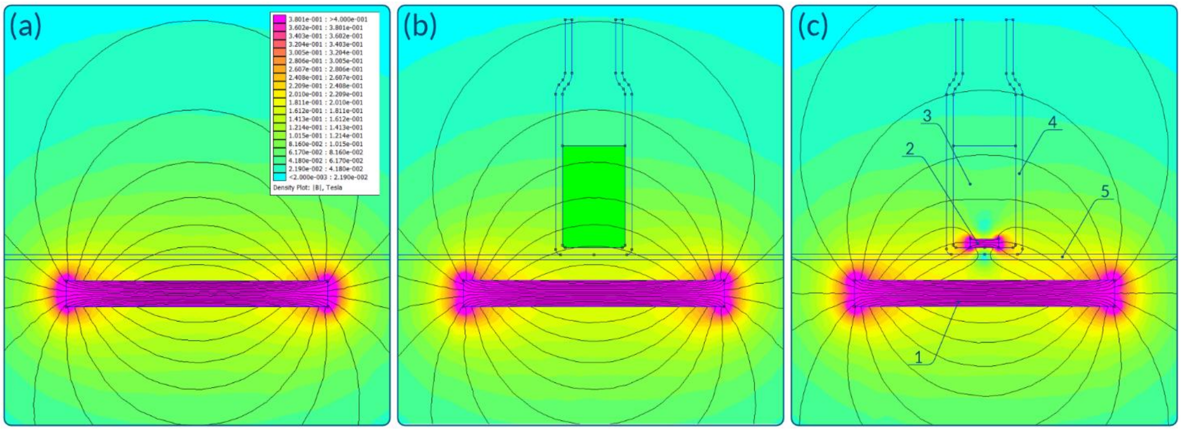

Figure S15. Maps of the flux density B (Tesla) calculated by the finite element method for different systems: (a) stirrer under-plate magnet and aluminum plate; (b) stirrer under-plate magnet, aluminum plate and glass vial with chloroform; (c) stirrer under-plate magnet, aluminum plate, glass vial with chloroform and stir bar. 1 – stirrer under-plate magnet, 2 – stir bar magnet, 3 – chloroform, 4 – glass vial, 5 – aluminum plate.

COMPARISON OF DIFFERENT STIR BAR SIZES AND SHAPES

Decomposition of the $Pd_2dba_3{\cdot}CHCl_3$ complex using stir bars of different sizes. 3 mg of $Pd_2dba_3{\cdot}CHCl_3$ was dissolved in 24 ml of $CHCl_3$, and then 8 ml of solution was added to each vial with different bars: an ISOLAB PTFE cylindrical stirrer bar of 15 × 6 mm, a VWR® PTFE cylindrical stirrer bar of 10 × 3 mm, and an LLG® PTFE cylindrical stirrer bar of 10 × 6 mm. Complex decomposition was carried out at room temperature using a Heidolph MR Hei-Tec stirrer at 500 rpm. A specially designed stand was installed on its surface (Figure S16), utilizing the three central positions for vessel placement. Complex decomposition was detected by a color change using time-lapse photography with a Nikon D610 camera with a Nikon AF-S Nikkor 35 mm lens.

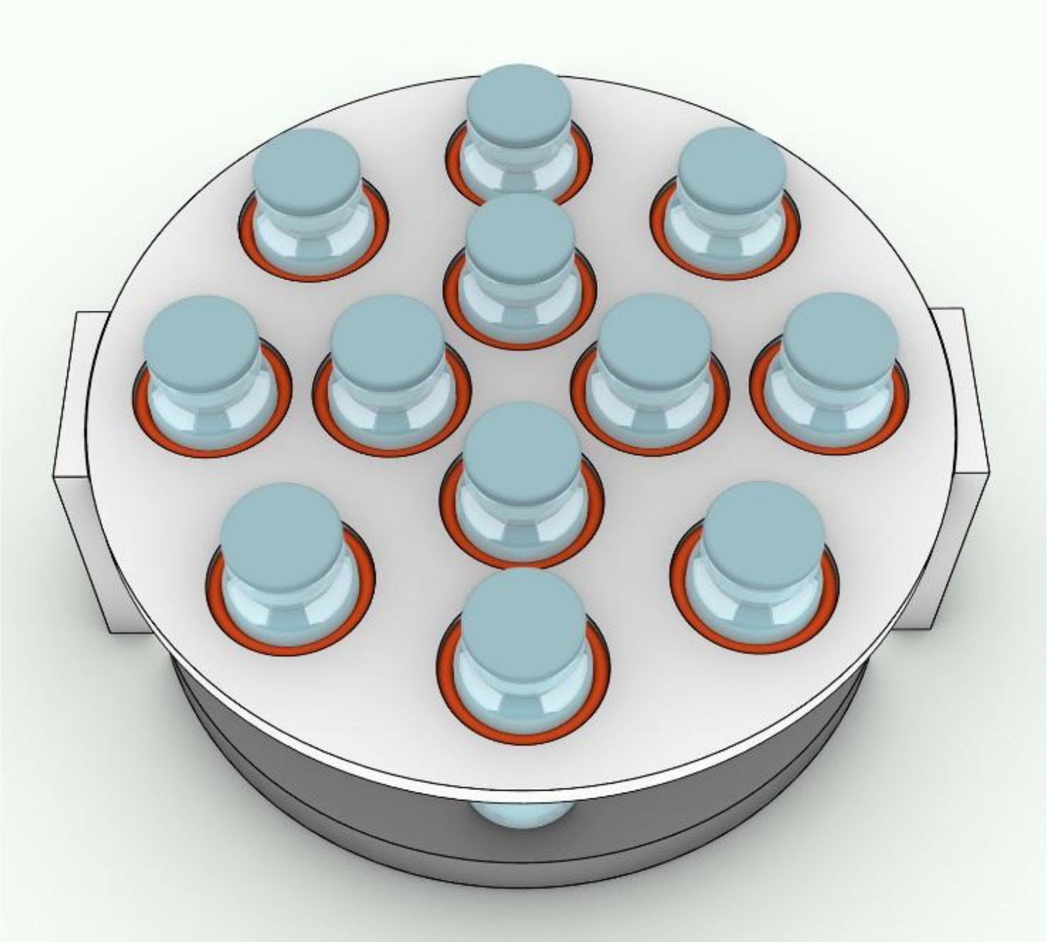

Figure S16. 3D model of the custom stand assembly showing vial positioning on the magnetic stirrer**.**

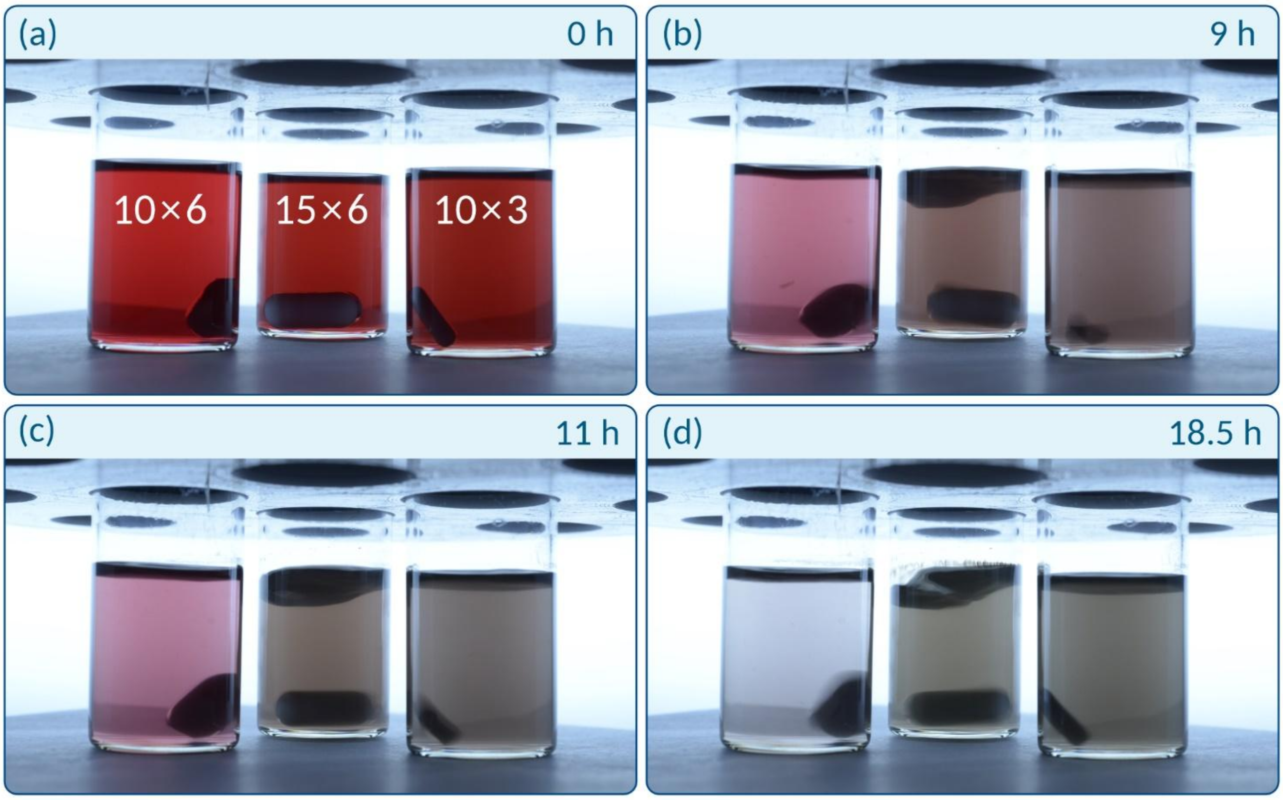

Figure S17. Time-lapse frames showing $Pd_2dba_3 \cdot CHCl_3$ complex decomposition using different stir bar sizes: (left) 10 × 6 mm, (center) 15 × 6 mm, (right) 10 × 3 mm.

The solution on the right undergoes discoloration first, followed by the center solution. The solution on the left undergoes discoloration last (Figure S17).

For comparative analysis of different stir bar motion dynamics with changing distance from the stirrer center, the following parameters were selected: VWR® PTFE cylindrical stirrer bar, 10 × 3 mm; VWR® PTFE cylindrical stirrer bar, 8 × 3 mm; SYNTHWARE GLASS SC0813 octagon cross-section, PTFE, C 8 ×13 mm; IsoLab oval, PTFE, 20 × 8 mm; SYNTHWARE GLASS SA0610 egg-shaped, PTFE, A 6 × 10 mm; and several unbranded commercial stir bars.

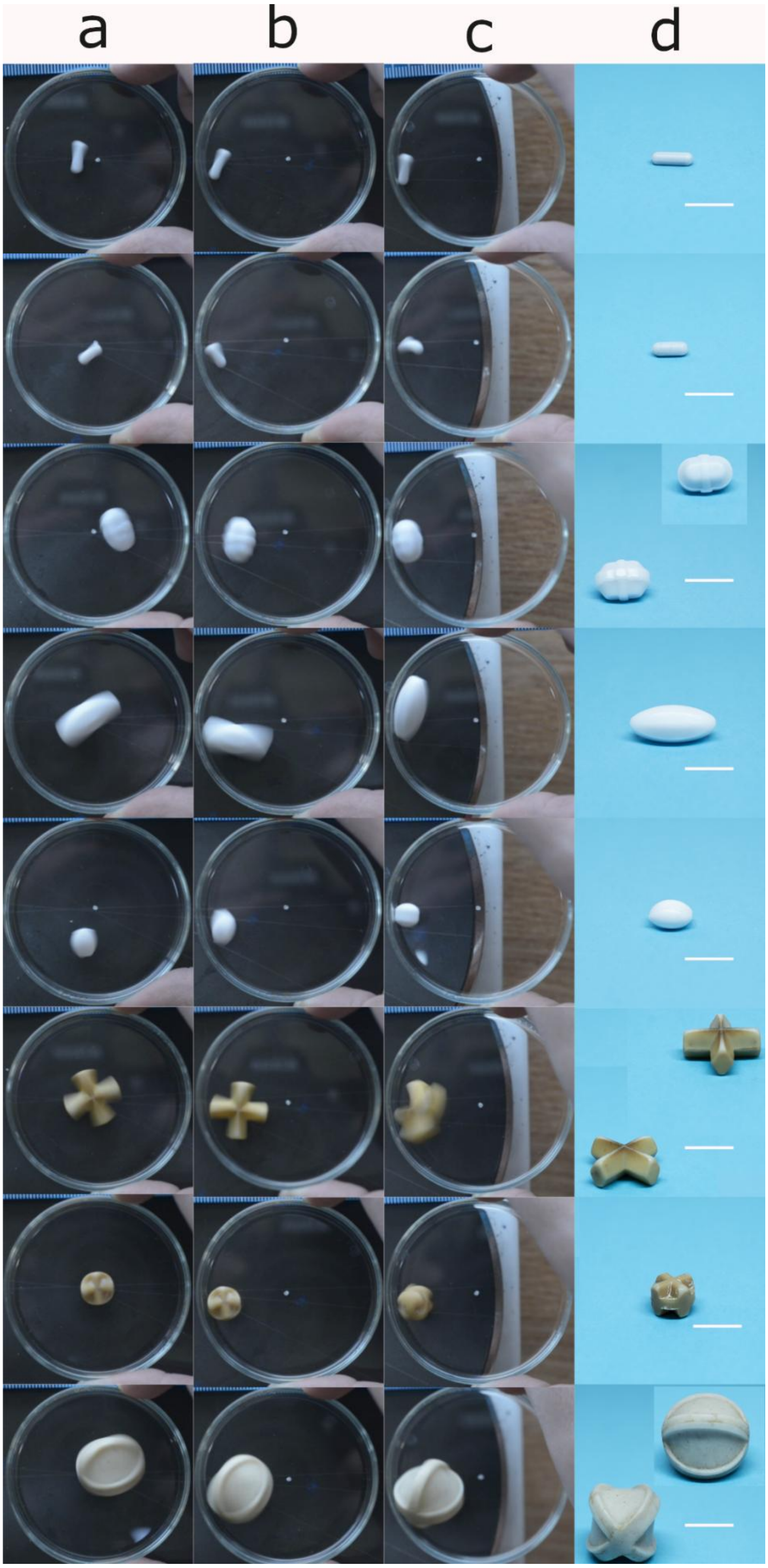

Figure S18. Rotation behavior of stir bars at various distances from the center (a-c) and physical images of the bars (d). Scale bar: 10 mm.

MICROSCOPY STUDY OF Pd NANOPARTICLES DEPOSITED ON NANOTUBES

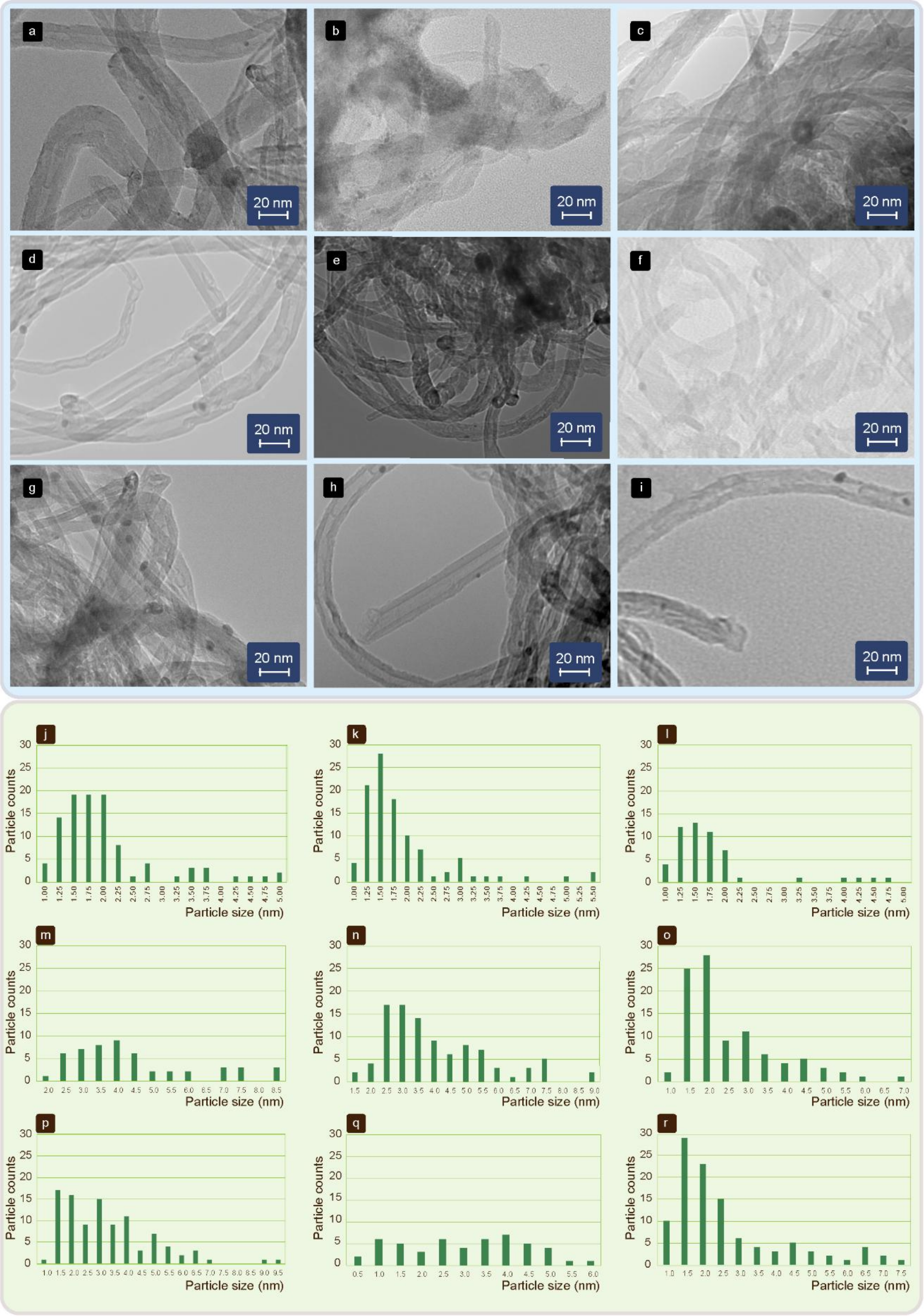

Figure S19. (a-i) TEM images of Pd nanoparticles deposited on nanotubes; (j-r) – particle size distribution; (a, j) – position 3a; (b, k) – position 3b; (c, l) – position 3c; (d, m) – position 2a; (e, n) – position 2b; (f, o) – position 2c; (g, p) – position 1a; (h, q) – position 1b; (i, r) – position 1c.

MICROSCOPY STUDY OF NANOTUBE CONGLOMERATES

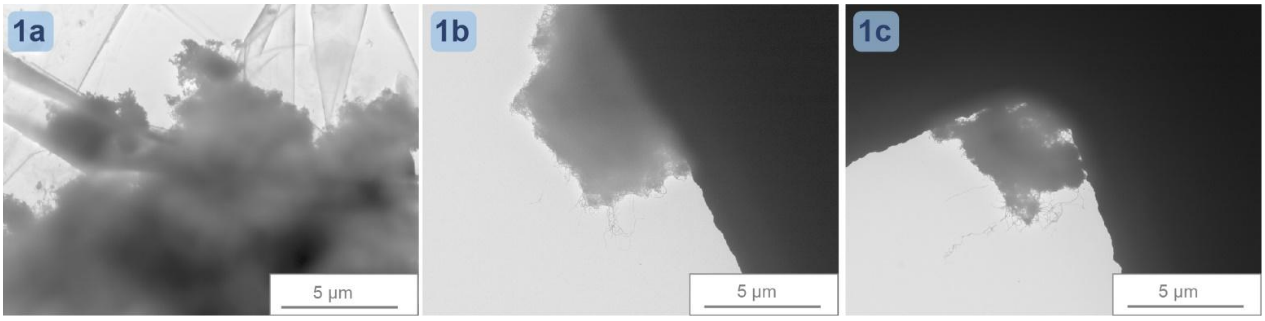

Figure S20. TEM images of nanotube conglomerates in vials at different positions (1a, 1b, and 1c), where 1a corresponds to the central position, while 1b and 1c are closer to the edge. Comparative analysis of these images reveals distinct differences in conglomerate sizes across positions.

REPRODUCIBILITY SCREENING IN DIFFERENT REACTIONS

Cu/C catalyst preparation. 15 mg of carbon nanotubes were dispersed in 3 ml of ethanol in each reaction vessel. 0.1 M copper(II) acetate solution in ethanol was prepared, and 200 µl of this solution was added dropwise to the nanotube suspension under continuous magnetic stirring at 500 rpm and room temperature. The setup shown in Figure 5b was used. After the mixture was allowed to stir for one hour, the system was heated to 70 °C, at which point 20 µl of hydrazine hydrate was injected. The reaction was maintained at 70 °C for two hours, with uniform heating in a water bath provided by two mechanical stirrers, IKA Overhead Stirrer RW11. The resulting Cu/C catalyst was thoroughly washed three times with deionized water and absolute ethanol to remove any residual reagents and then dried under vacuum. The catalyst morphology and copper nanoparticle distribution were characterized *via* transmission electron microscopy (TEM).

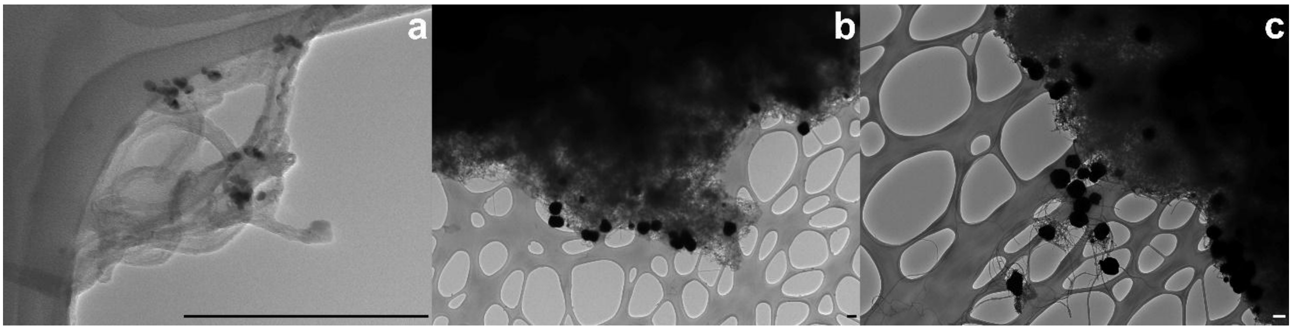

Figure S21. TEM images of Cu on nanotubes in different vial positions: (a) center, (b) adjacent to center, (c) edge. Scale bar: 200 nm.

Table S2. Position-dependent average particle dimensions.

**Position**	**a**	**b**	**c**
**Average particle size, nm**	5.4±3.1	9.2±1.7<br>196.7±68.8	32.9±10.6<br>180.6±68.5

In off-central positions, two distinct particle types were observed: small and large. Notably, the size of the small particles varied across all three positions.

Belousov–Zhabotinsky reaction (oscillatory)

Preparation of the Ferroin Solution

Ferroin indicator was prepared by dissolving 0.7 g of $FeSO_4·7H_2O$ in 90 ml of deionized water, followed by the addition of 5 g of *o*-phenanthroline. The mixture was stirred until complete dissolution and subsequently diluted to a final volume of 100 ml with deionized water.

Preparation of reaction solutions

Three separate stock solutions were prepared:

1. 2.78 g of potassium bromate was dissolved in 33.5 ml of deionized water and acidified with 1 ml of concentrated sulfuric acid;
2. 0.5 g of malonic acid was dissolved in 5 ml of deionized water;
3. 0.5 g of potassium bromide was dissolved in 5 ml of deionized water.

Reaction Setup

Each reaction vessel received 3 ml of oxidizing solution (1), 0.5 ml of malonic acid solution (2), and 0.25 ml of potassium bromide solution (3). The reactions were initiated by the simultaneous addition of 0.5 ml of ferroin solution to all six vessels. The reaction systems were arranged on a two-tier stand and monitored for 20 minutes.

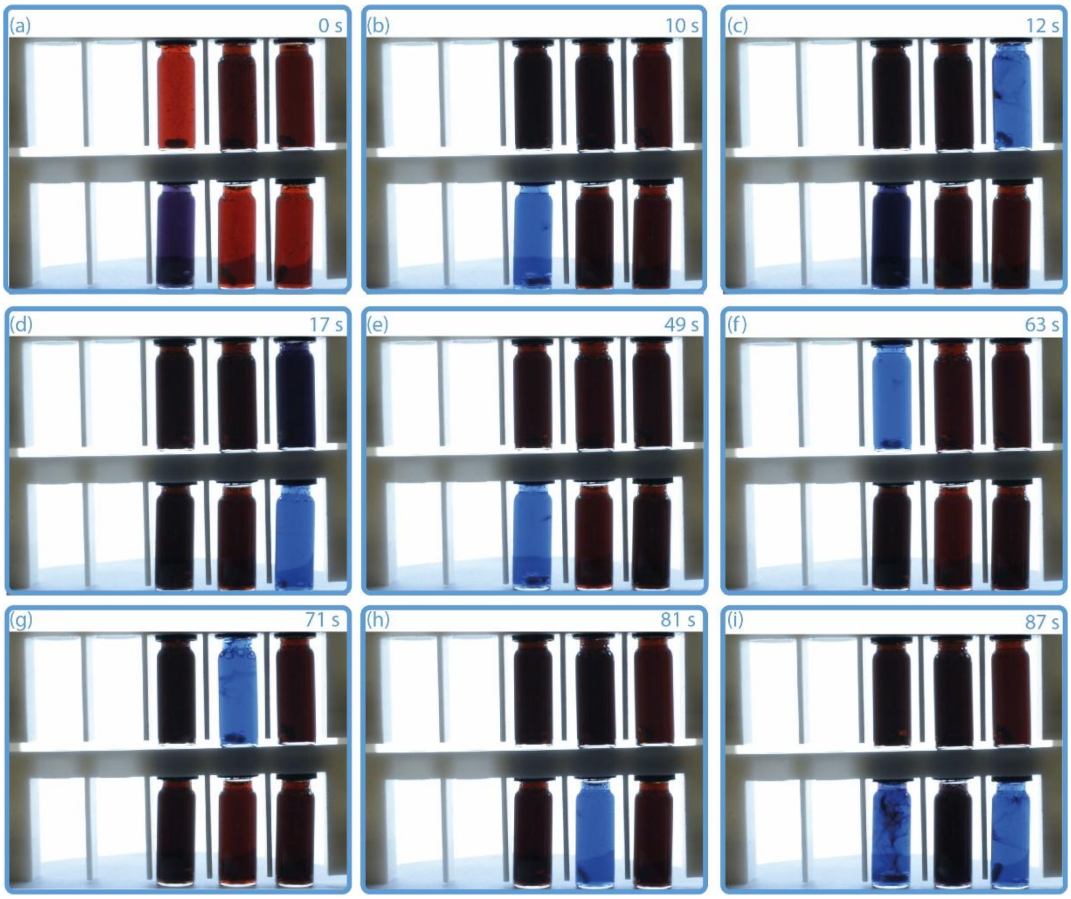

Figure S22. Monitoring frames of the oscillatory reaction.

During the observation period (20 minutes), the color of the solution in the central vial at the bottom changed 25 times, whereas the central vial at the top exhibited 20 transitions. For the two solutions in non-central positions on the first tier, 8 color changes were observed each. On the second tier, the solution in the second position from the center showed 16 transitions, whereas the outermost one displayed 10.

Polymerization

1 wt.% of azobisisobutyronitrile (AIBN) was added to 2 ml of methyl acrylate in each vessel. The solution was blown through with argon for five minutes and then heated to 90 °C under stirring.

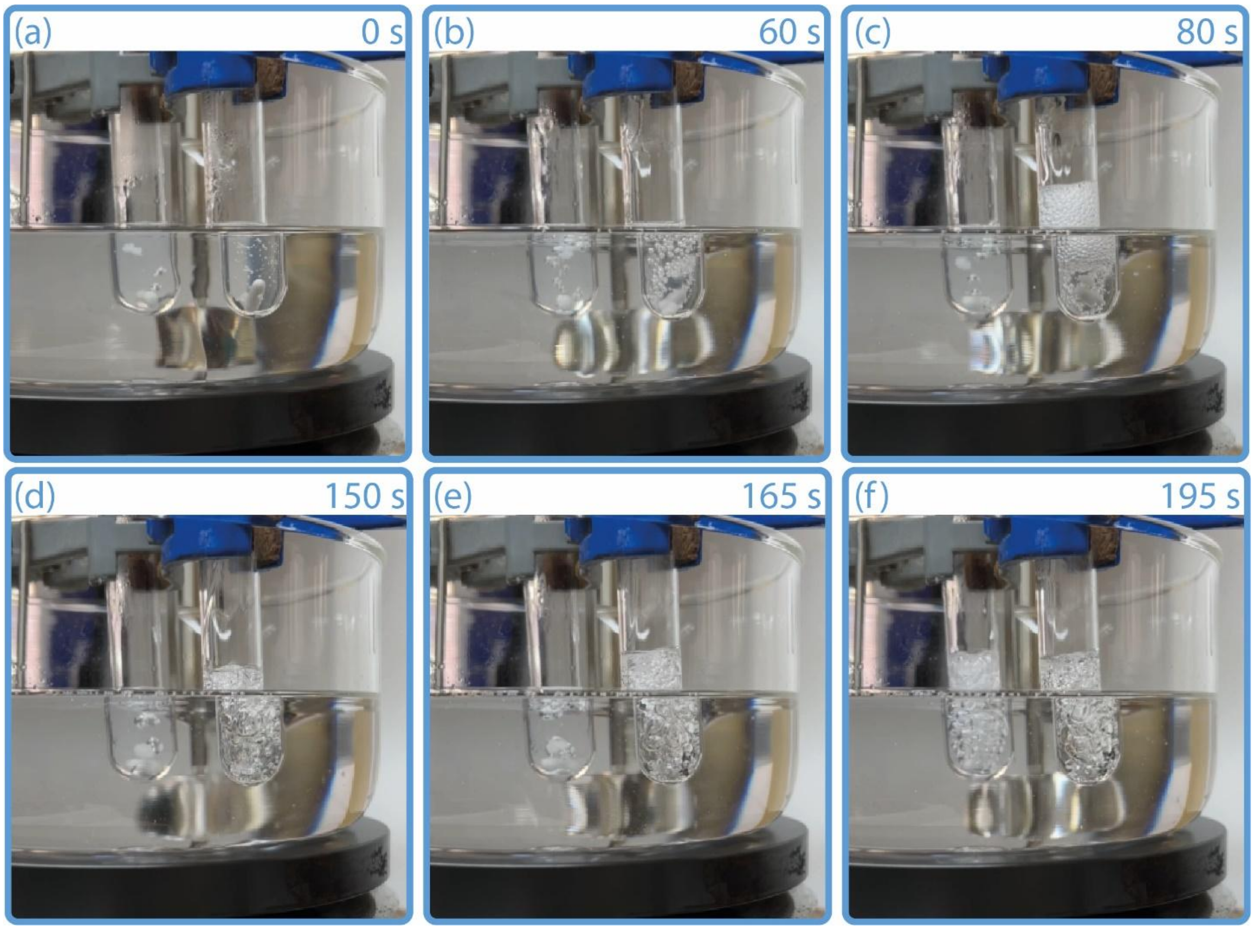

Figure S23. Monitoring frames of the polymerization.

Real-time monitoring revealed significantly accelerated process development in off-center vessels, as evidenced by the sequential cessation of stirring, volumetric expansion, and rapid gel formation. This spatial heterogeneity in reaction progress may originate from localized thermal

gradients caused by suboptimal convective mixing, promoting faster initiator decomposition and chain propagation.

EJECTION OF REAGENTS FROM THE REACTION SYSTEM DUE TO STIRRING

Decomposition of the $Pd_2dba_3{\cdot}CHCl_3$ complex on carbon black

1.5 mg of $Pd_2dba_3{\cdot}CHCl_3$ was dissolved in 12 ml of $CHCl_3$, and then 2 ml of solution and 20 mg of carbon black was added to each vial (Microlab Scientific, color clear, vial volume 4 ml, cap type: 13-425, 15 × 45 mm) with bars (SYNTHWARE GLASS SA0610 egg-shaped, PTFE, A 6 × 10 mm). Complex decomposition was carried out at room temperature using a Heidolph MR Hei-Tec stirrer at 500 rpm. A specially designed stand was installed on its surface (Figure 2b). Complex decomposition was detected visually by a color change. The decomposition process was recorded using time-lapse photography with a Nikon D610 camera with a Nikon AF-S Nikkor 35 mm lens.

In vials with off-center positioning, the ejection of carbon material is observed within five minutes of reaction initiation. The expelled material quantity subsequently increases until it reaches a steady-state level.

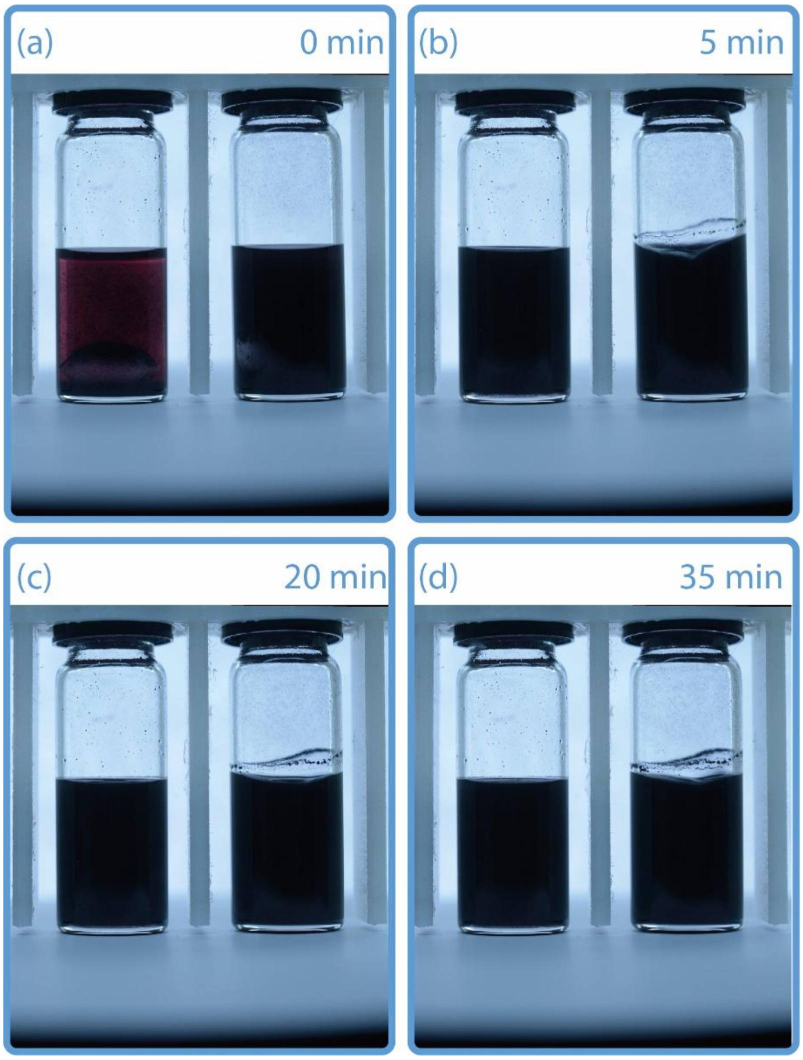

Figure S24. Time-lapse video frames showing $Pd_2dba_3 \cdot CHCl_3$ complex decomposition. Left – center position, right – off-center position.

MAGNETIC BAR MOTION DYNAMICS

In order to demonstrate the bar motion dynamics over different vials, the vials were recorded on video. The recording process was carried out from above through the necks of the vials. The footage included the bar acceleration and the stationary mode of rotation. For clarity and ease of presentation, the video frames were divided into stacks of 100 and superimposed on top of each other. Thus, one frame shows the total positions of the bar for 1.67 seconds.

Table S3. Cumulative representation of bar location in **1a** position.

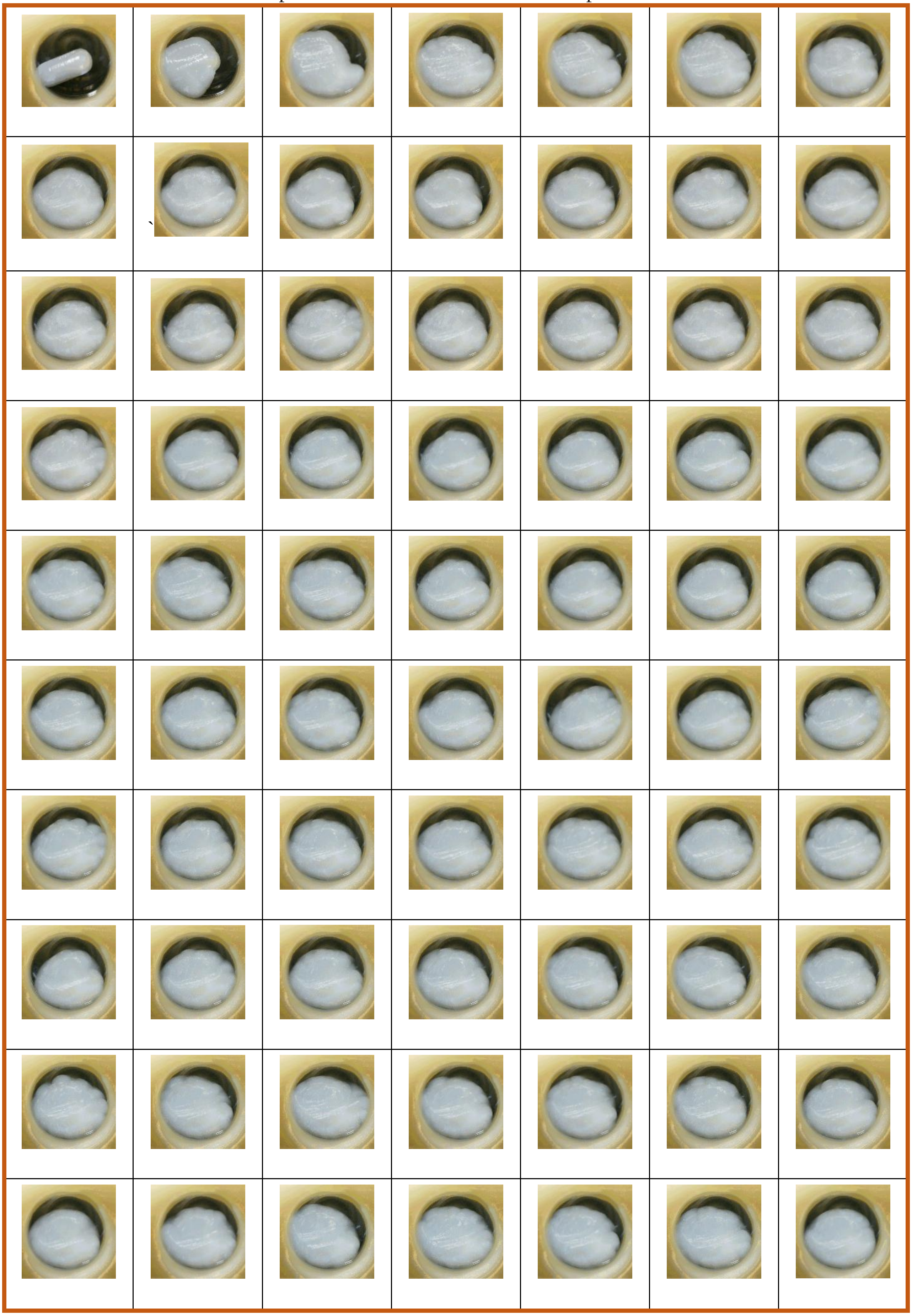

Table S4. Cumulative representation of bar location in **1b** position.

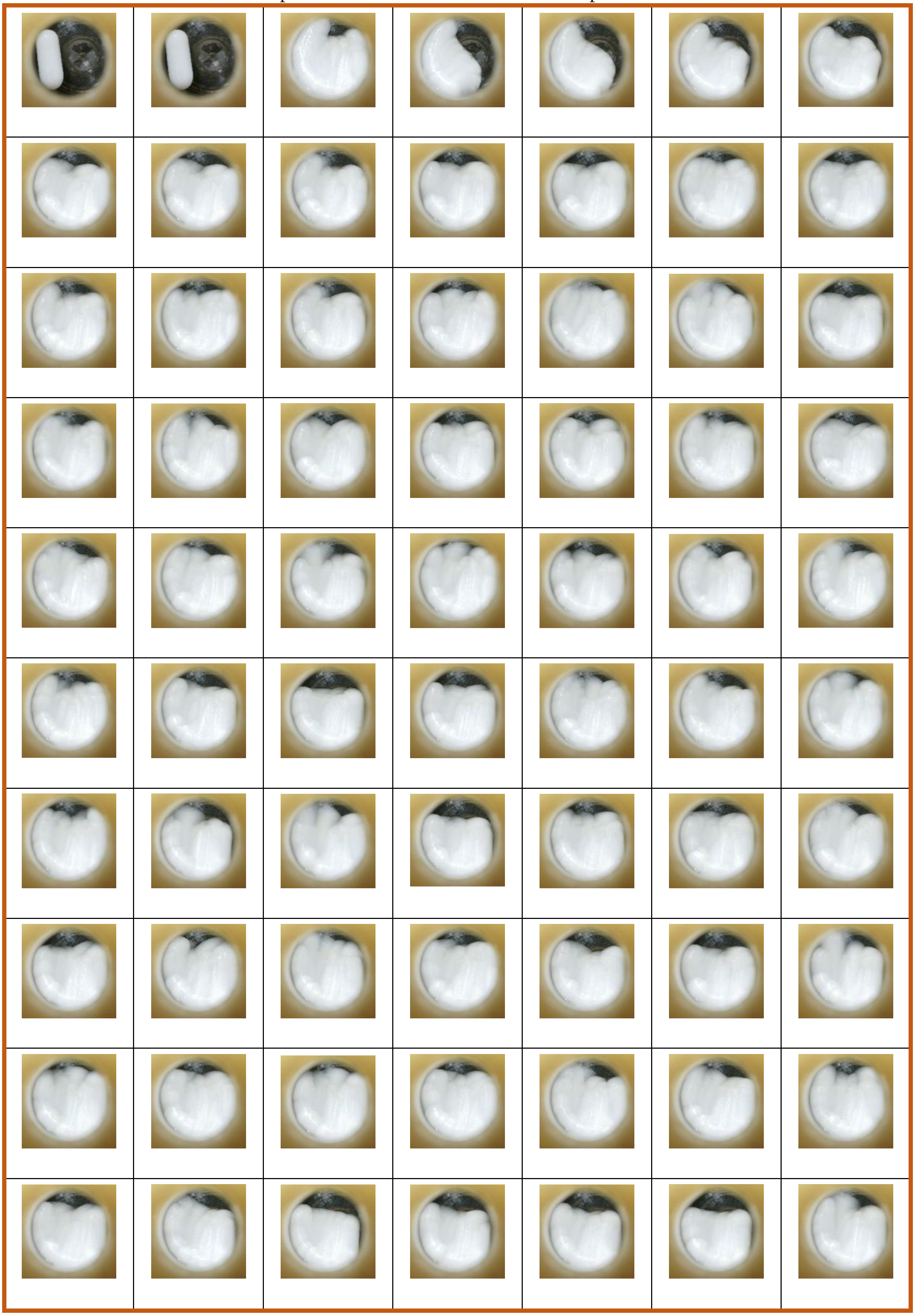

Table S5. Cumulative representation of bar location in **1c** position.

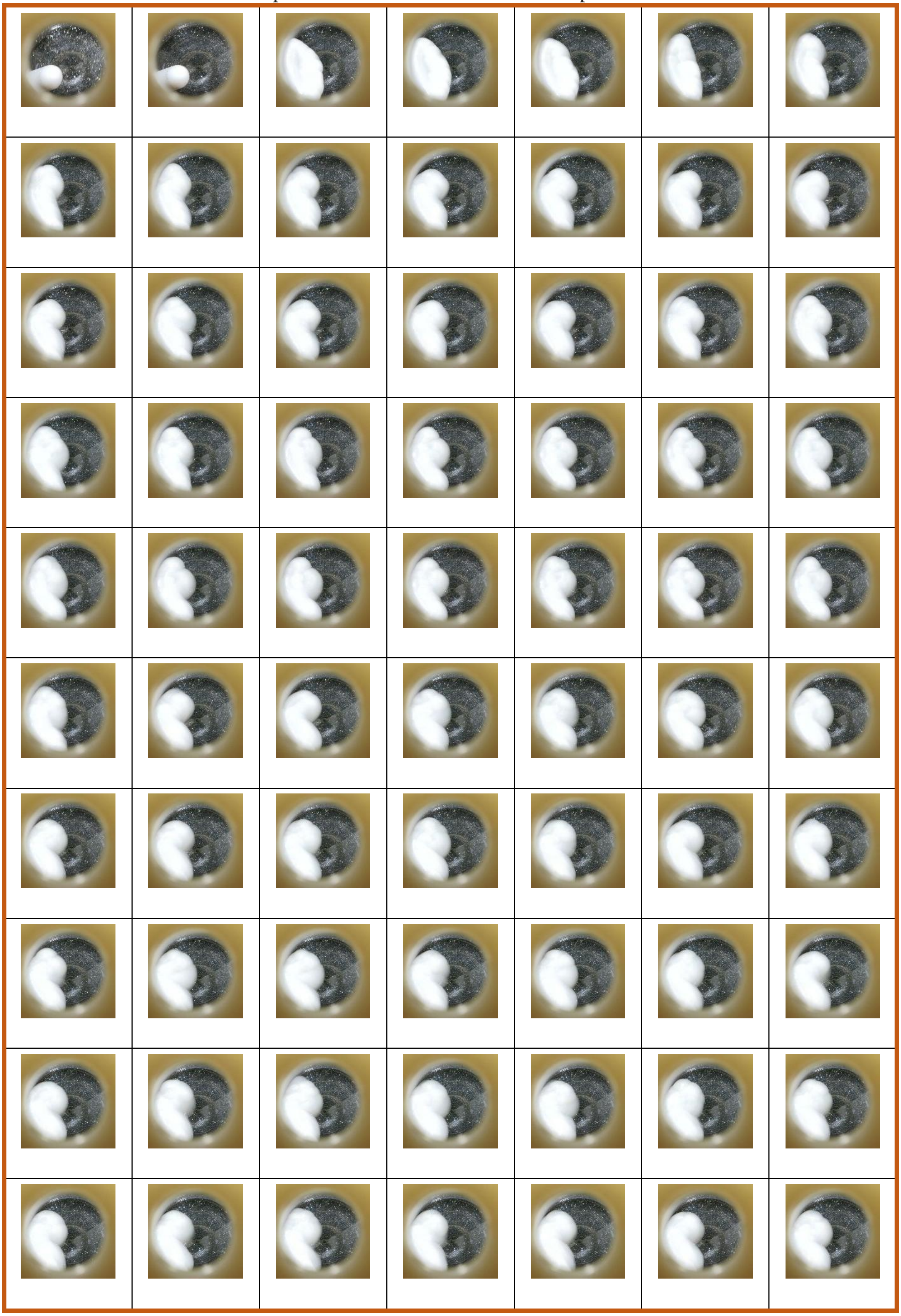

Table S6. Cumulative representation of bar location in **2a** position.

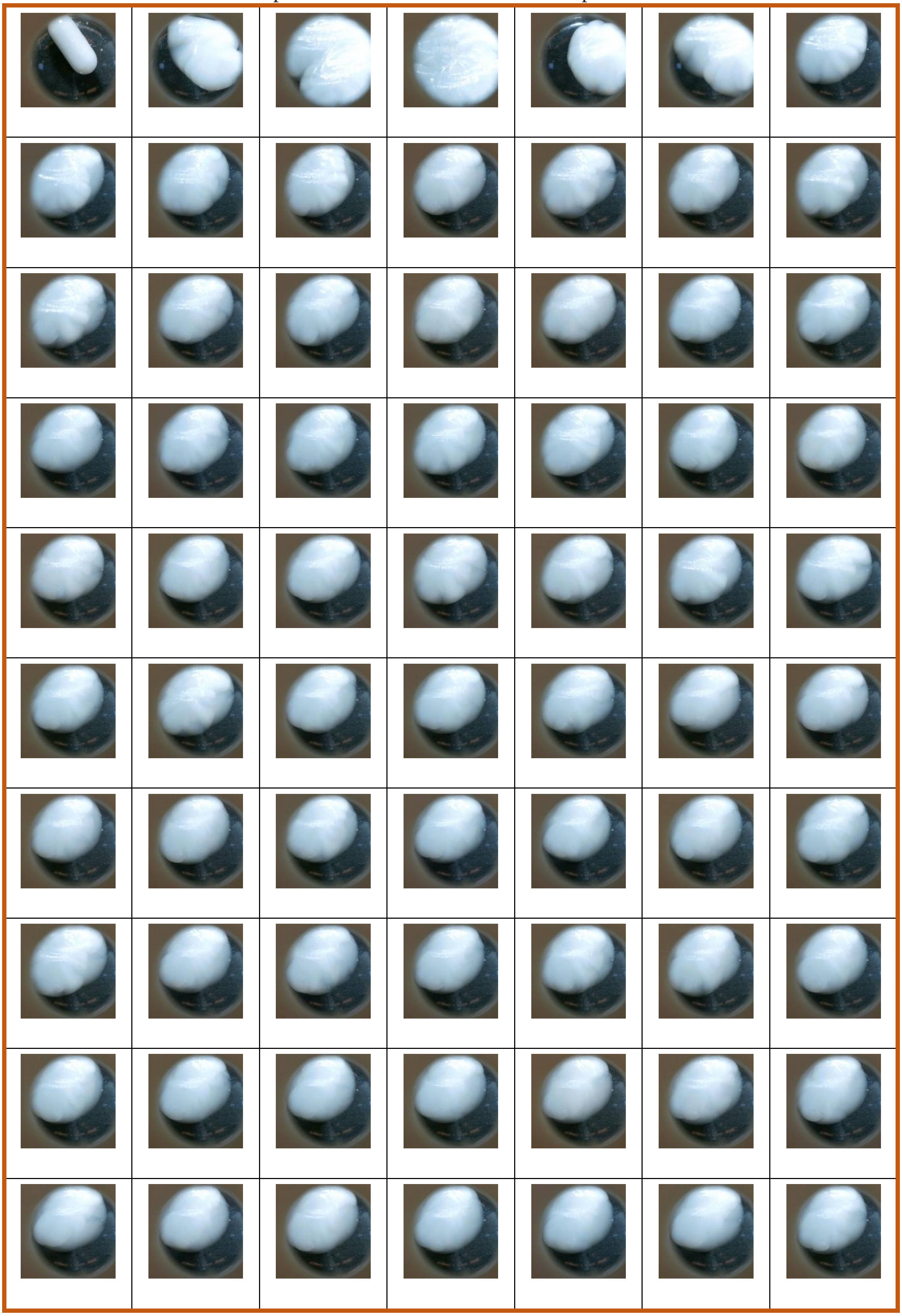

Table S7. Cumulative representation of bar location in **2b** position.

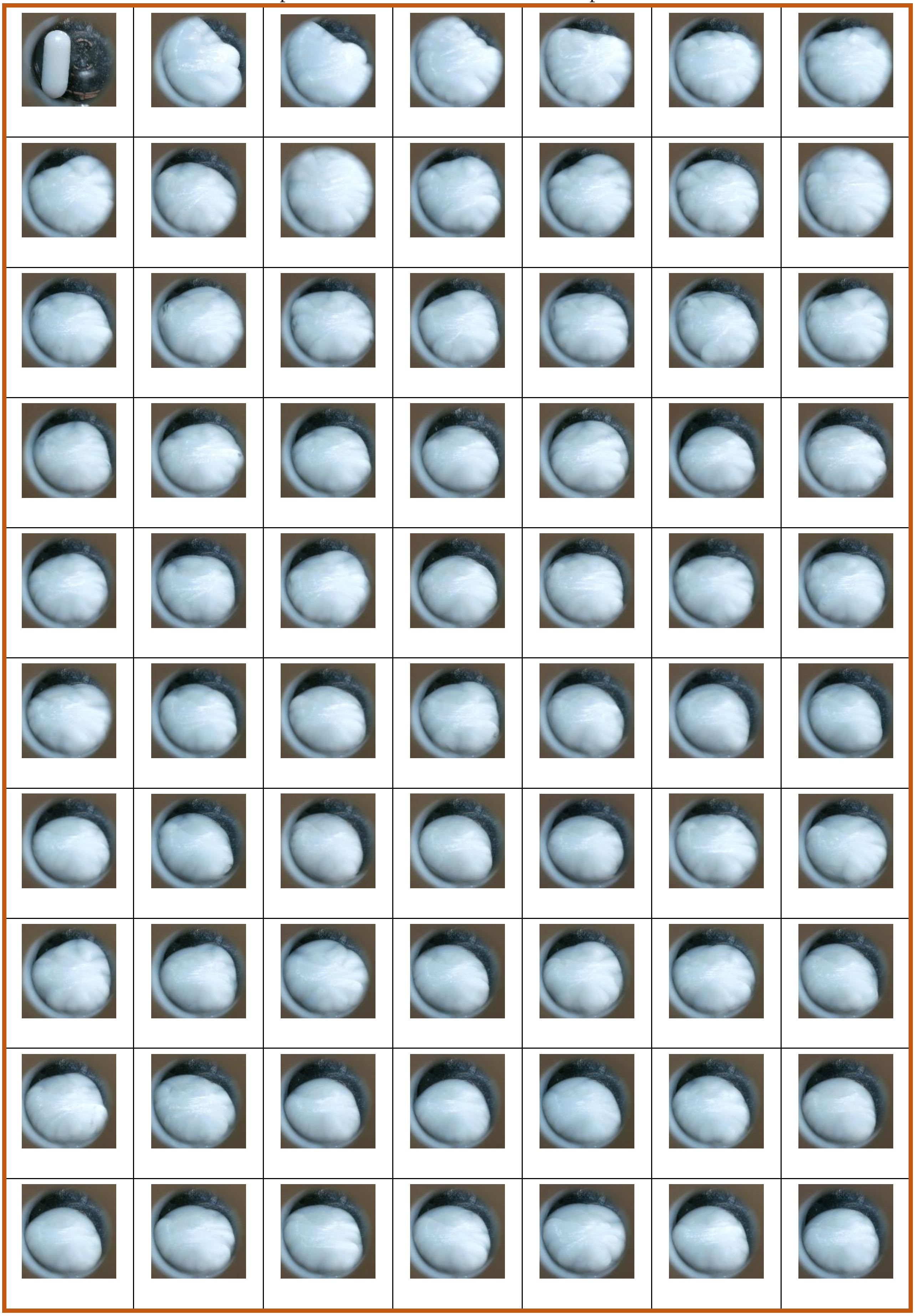

Table S8. Cumulative representation of bar location in **2c** position.

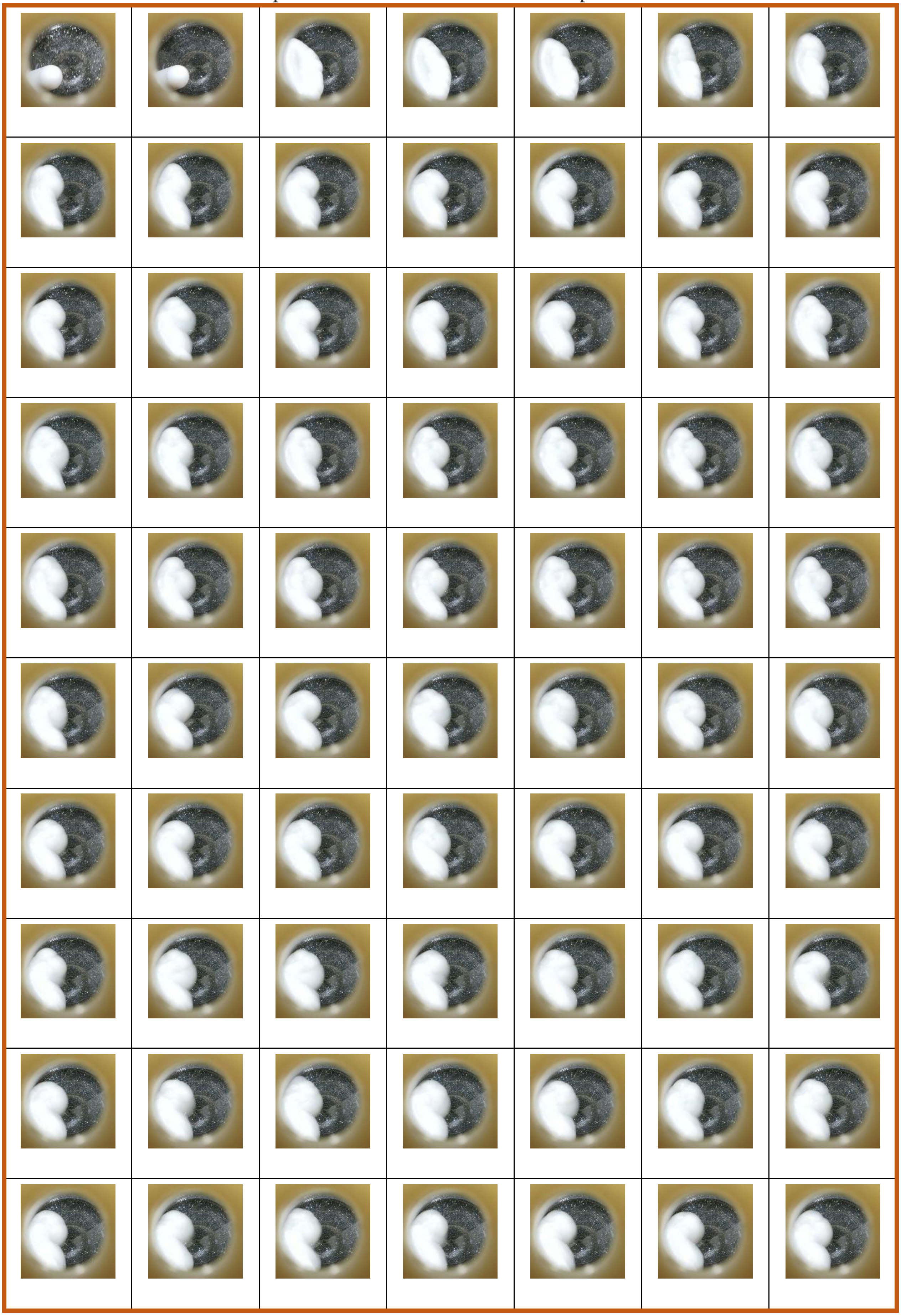

Table S9. Cumulative representation of bar location in **3a** position.

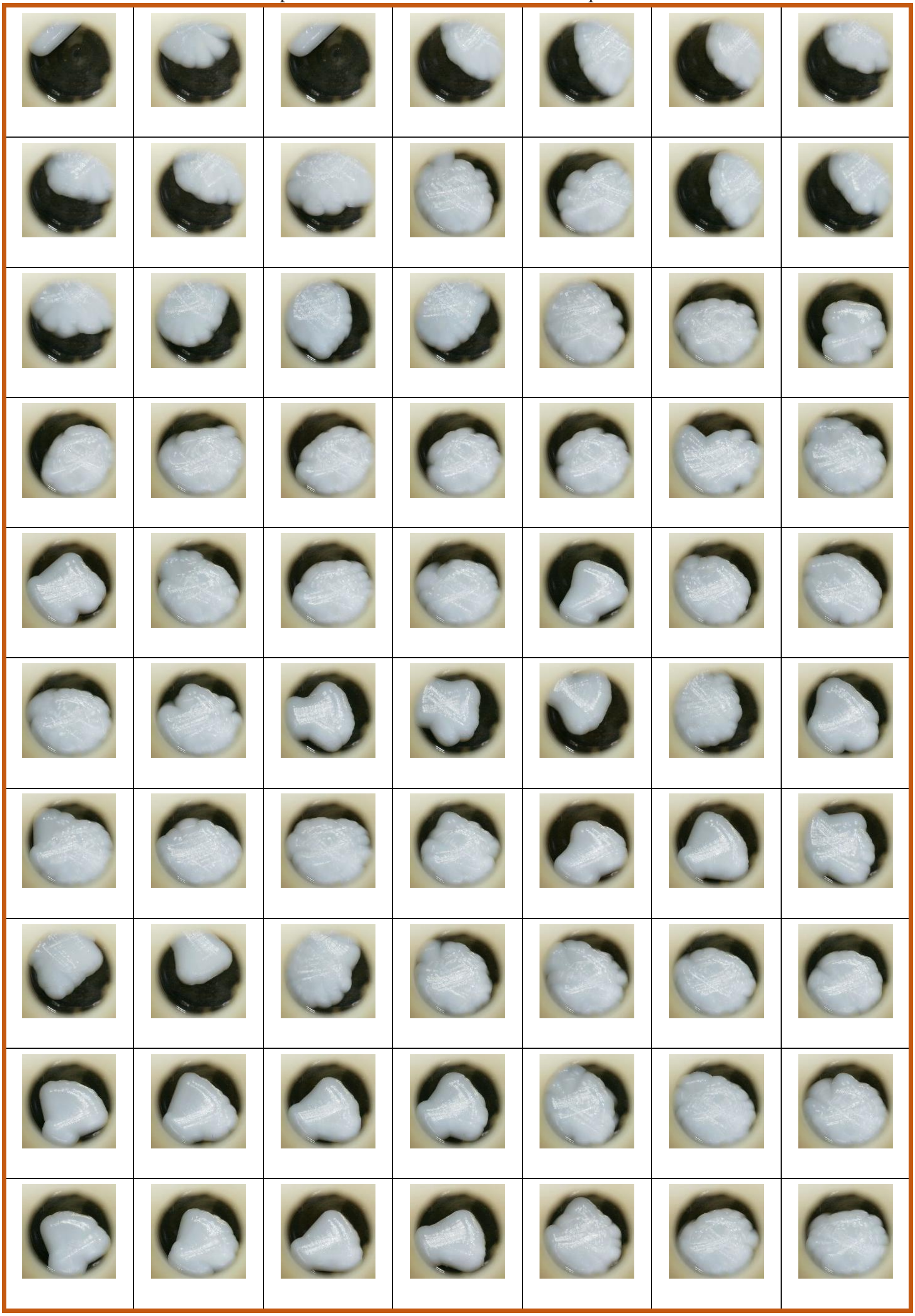

Table S10. Cumulative representation of bar location in **3b** position.

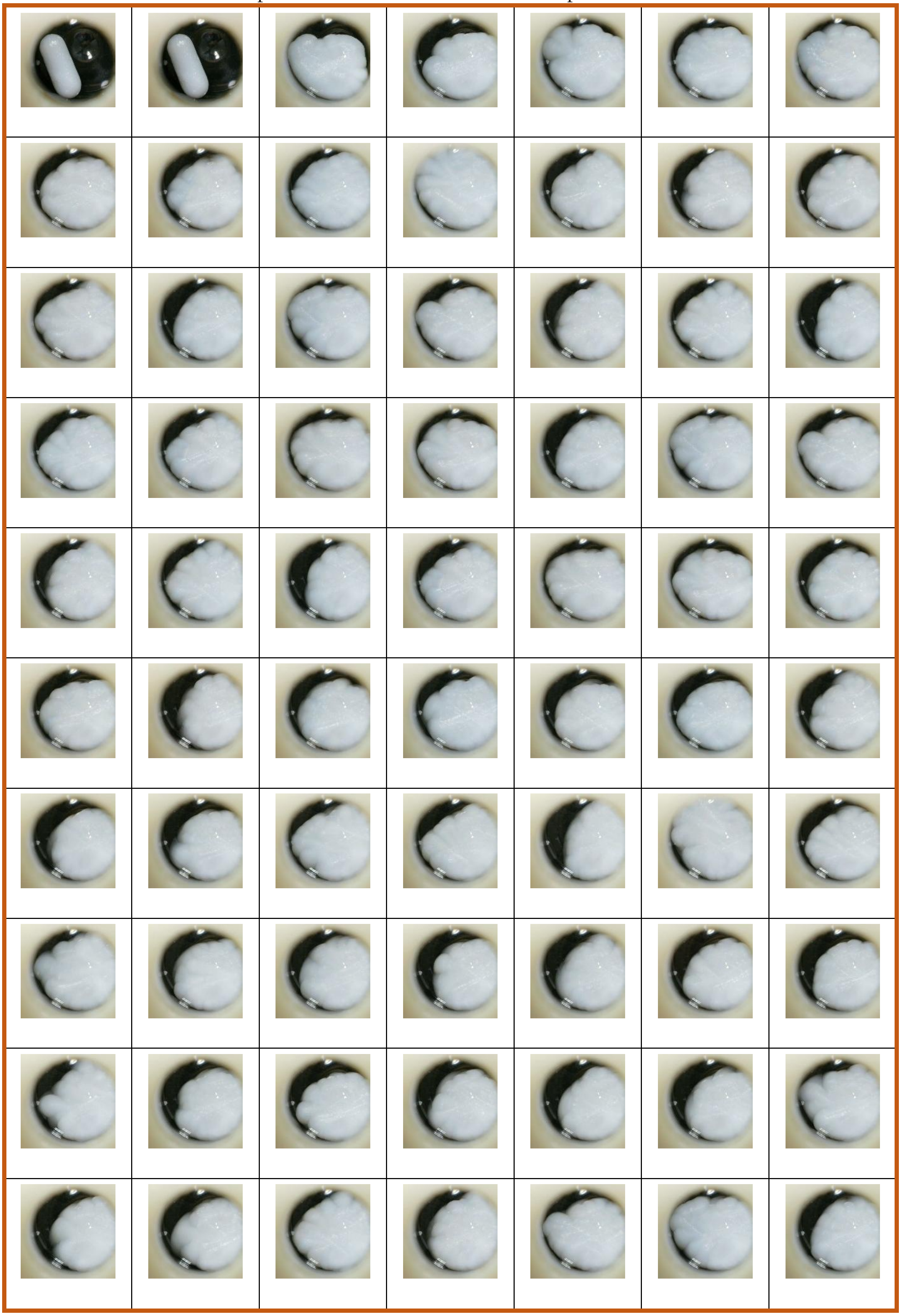

Table S11. Cumulative representation of bar location in **3c** position.

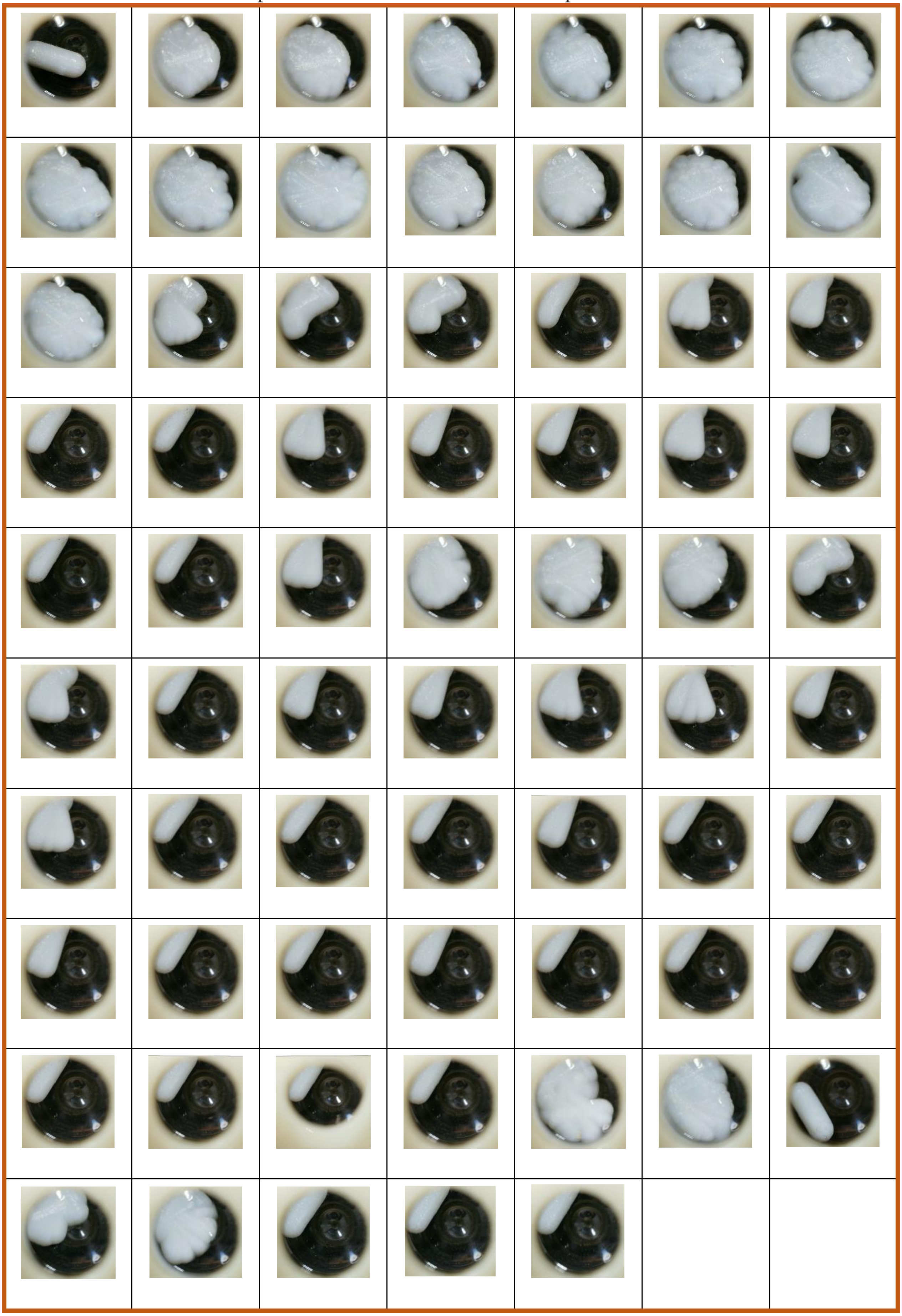

REACTIONS IN DIFFERENT VESSELS

Decomposition of the $Pd_2dba_3{\cdot}CHCl_3$ complex in different vessels

1.5 mg of $Pd_2dba_3{\cdot}CHCl_3$ was dissolved in 12 ml of $CHCl_3$, and then 3.95 ml of solution was added to three different vessels (round-bottom flask, conical flask, cylindrical vial). Complex decomposition was carried out at room temperature using a Heidolph MR Hei-Tec stirrer at 500 rpm. The centers of the vessels were fixed at equal distances from the stirrer center. Complex decomposition was detected visually by a color change. The decomposition process was recorded *via* time-lapse photography with a Nikon D610 camera with a Nikon AF-S Nikkor 35 mm lens.

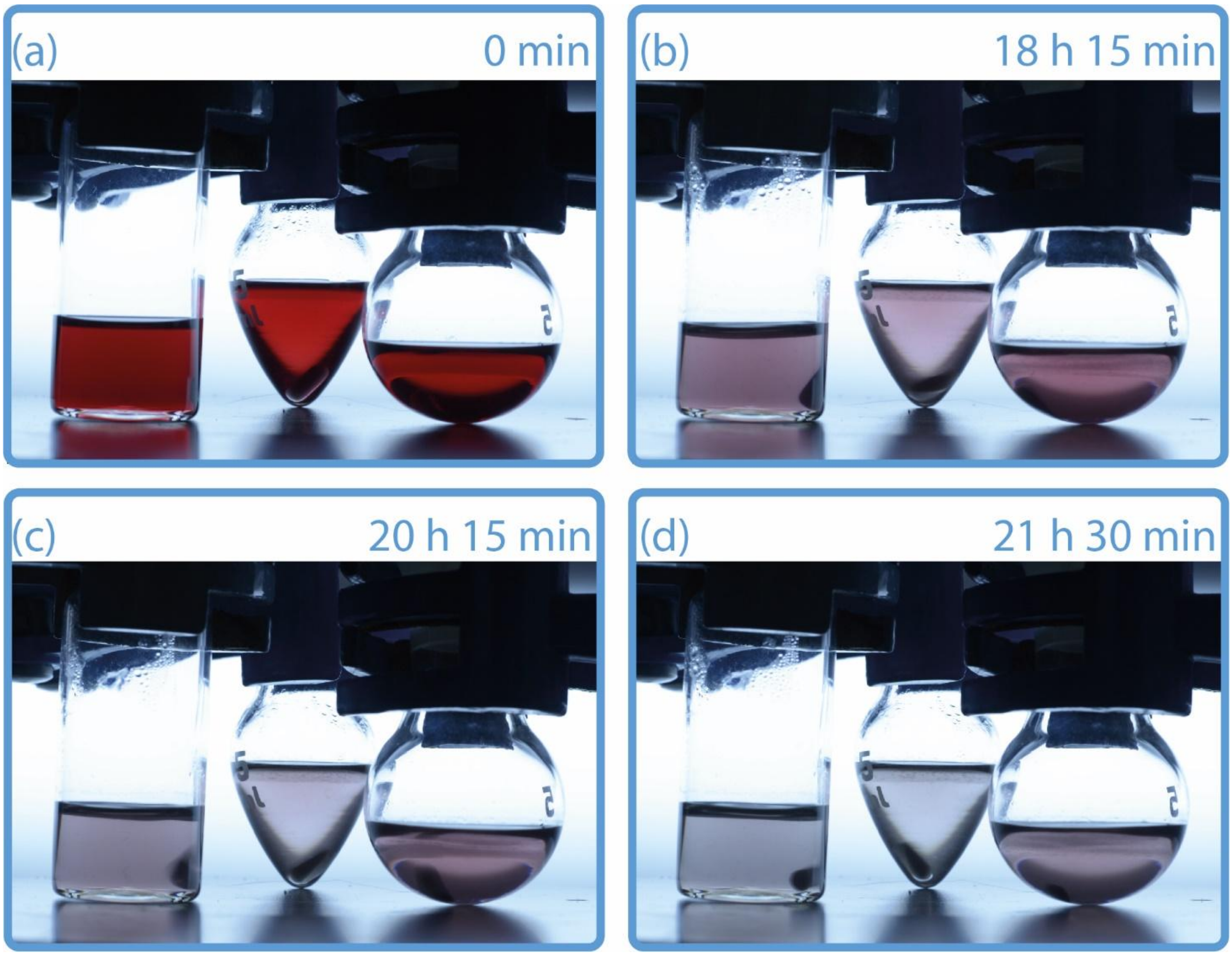

Figure S25. Time-lapse video frames showing the $Pd_2dba_3{\cdot}CHCl_3$ complex in vessels of different shapes.

VISUALIZATION OF THE MAGNETIC FIELD EFFECT

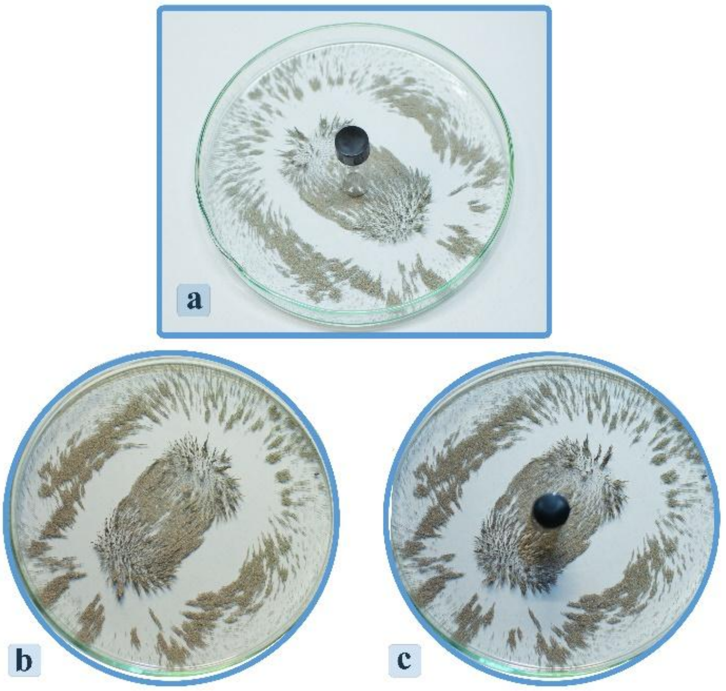

Figure S26. Magnetic field visualization with an empty vial. (a) – Side view, (b) – visualization without a vial, (c) – visualization with a vial.

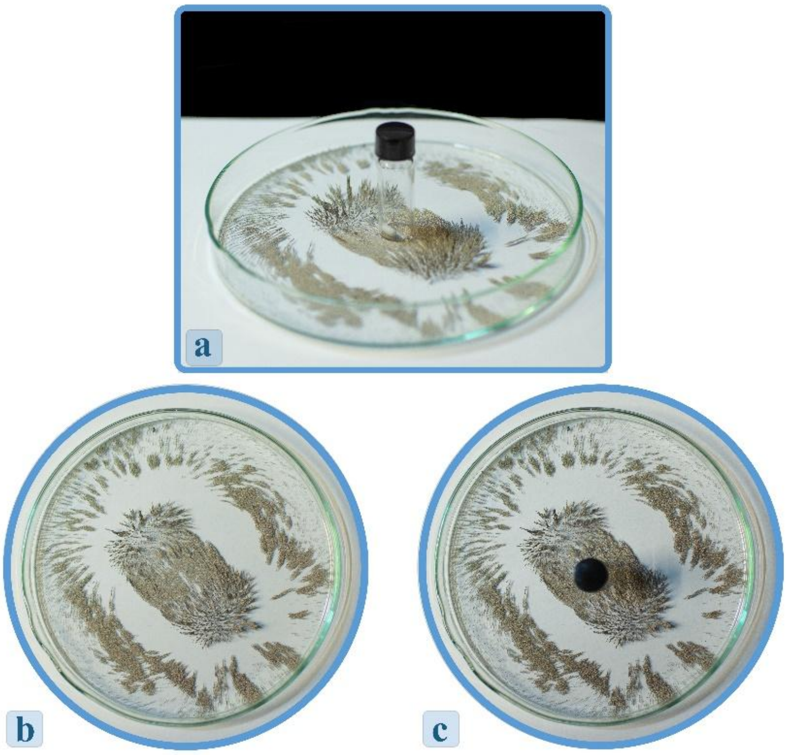

Figure S27. Magnetic field visualization with a bar in a vial. (a) – Side view, (b) – visualization without a vial, (c) – visualization with a vial and a bar.

Several magnetic stirrers from different manufacturers were selected to illustrate the differences in the magnetic fields of various stirrers. Equal amounts of nickel granules were placed on each stirrer and covered with a sheet of paper for comparison.

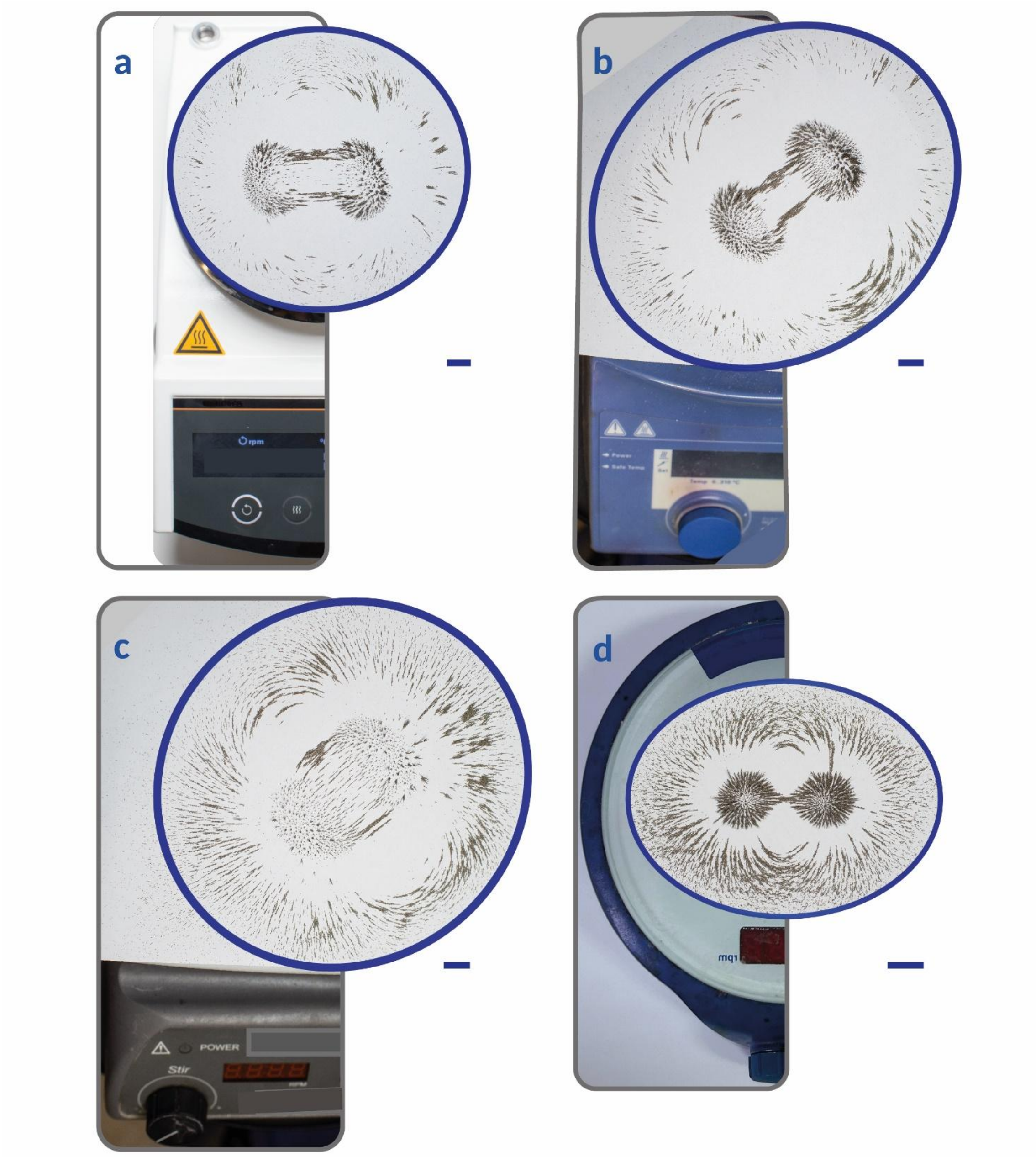

Figure S28. Images of magnetic stirrers and visualization of their magnetic fields using magnetic nickel granules. (a) – Corresponds to stirrer model 1 (Figure 6b), (b) – corresponds to stirrer model 2 (Figure 6c), (c) – corresponds to stirrer model 4 (Figure 6e), and (d) – corresponds to stirrer model 5 (Figure 6f). The scale bar corresponds to 1 cm.

VISUALIZATION WITH SILICONE-BASED FERROFLUID

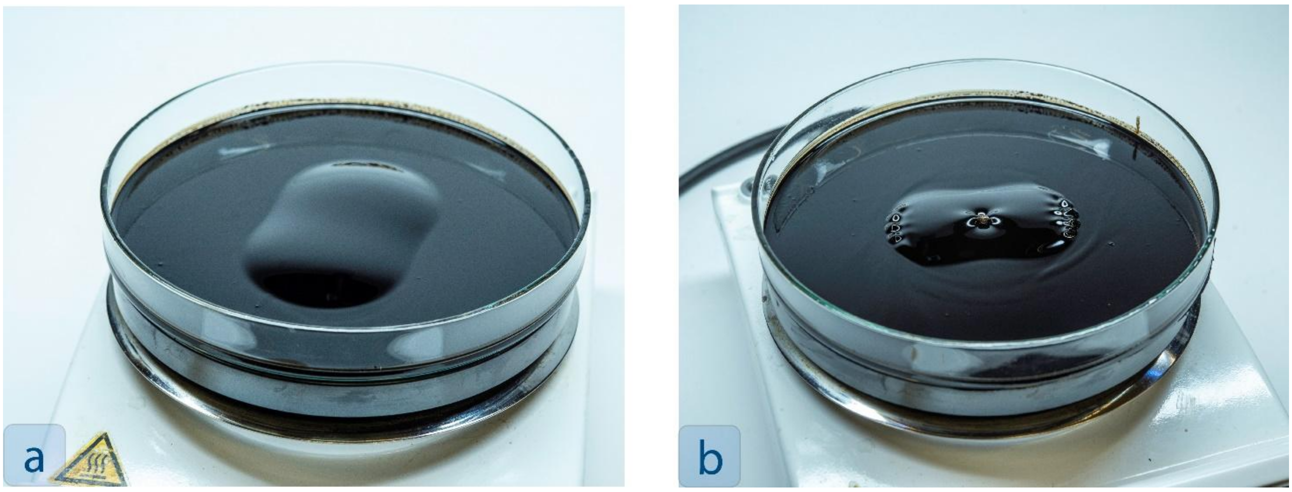

Figure S29. Ferrofluid interaction with a magnetic field because of their magnetic susceptibility. The same amount of ferrofluid on a flat surface: (a) – without a bar, (b) – with a bar.

REFERENCES

[1] Zalesskiy, S. S.; Ananikov, V. P. $Pd_2(dba)_3$ as a Precursor of Soluble Metal Complexes and Nanoparticles: Determination of Palladium Active Species for Catalysis and Synthesis. *Organometallics* **2012**, *31* (6), 2302–2309. DOI: 10.1021/om201217r.

[2] Kachala, V. V.; Khemchyan, L. L.; Kashin, A. S.; Orlov, N. V.; Grachev, A. A.; Zalesskiy, S. S.; Ananikov, V. P. Target-oriented analysis of gaseous, liquid and solid chemical systems by mass spectrometry, nuclear magnetic resonance spectroscopy and electron microscopy. *Russ. Chem. Rev.* **2013**, *82* (7), 648–685. DOI: 10.1070/RC2013v082n07ABEH004413.

[3] Kashin, A. S.; Ananikov, V. P. A SEM study of nanosized metal films and metal nanoparticles obtained by magnetron sputtering. *Russ. Chem. Bull.* **2011**, *60* (12), 2602–2607. DOI: 10.1007/s11172-011-0399-x.

[4] Meeker, D. Finite Element Method Magnetics (FEMM 4.2). **2019**.
